# Non-local triple quantum dot thermometer based on Coulomb-coupled systems

**DOI:** 10.1038/s41598-022-19596-5

**Published:** 2022-09-23

**Authors:** Suraj G. Dhongade, Afreen A. Haque, Sayan Saha Roy, Aniket Singha

**Affiliations:** Department of Electronics and Electrical Communication Engineering, Indian Institute of Technology Kharagpur, Kharagpur, 721302 India

**Keywords:** Nanoscale devices, Other nanotechnology

## Abstract

Recent proposals towards non-local thermoelectric voltage-based thermometry, in the conventional dual quantum dot set-up, demand an asymmetric step-like system-to-reservoir coupling around the ground states for optimal operation (Physica E, 114, 113635, 2019). In addition to such demand for unrealistic coupling, the sensitivity in such a strategy also depends on the average measurement terminal temperature, which may result in erroneous temperature assessment. In this paper, we propose non-local current based thermometry in the dual dot set-up as a practical alternative and demonstrate that in the regime of high bias, the sensitivity remains robust against fluctuations of the measurement terminal temperature. Proceeding further, we propose a non-local triple quantum dot thermometer, that provides an enhanced sensitivity while bypassing the demand for unrealistic step-like system-to-reservoir coupling and being robust against fabrication induced variability in Coulomb coupling. In addition, we show that the heat extracted from (to) the target reservoir, in the triple dot design, can also be suppressed drastically by appropriate fabrication strategy, to prevent thermometry induced drift in reservoir temperature. The proposed triple dot setup thus offers a multitude of benefits and could potentially pave the path towards the practical realization and deployment of high-performance non-local “sub-Kelvin range” thermometers.

## Introduction

Nanoscale electrical thermometry in the cryogenic domain, particularly in the sub-Kelvin regime, has been one of the greatest engineering challenges in the current era. Device engineering with the ambition to couple system thermal parameters with electrically measurable quantities has been extremely challenging in nano-scale regime. In the recent era of nano-scale engineering, thermal manipulation of electron flow has manifested itself in the proposals of thermoelectric engines^1,2^, refrigerators^3^, rectifiers^4^ and transistors^5,6^. In addition, the possibility of *non-local* thermal control of electrical parameters has been also been proposed and demonstrated experimentally^7,8^. In the case of non-local thermal control, electrical parameters between two terminals are dictated by temperature of one or more remote reservoirs, which are spatially and electrically isolated from the path of current flow. The electrical and spatial isolation thus prohibits any exchange of electrons between the remote reservoir(s) and the current conduction track, while still permitting the reservoir(s) to act as the heat source (sink) via appropriate Coulomb coupling^8,9^.

Thus, non-local thermal manipulation of electronic flow mainly manifests itself in multi-terminal devices, where current/voltage between two terminals may be controlled via temperature-dependent stochastic fluctuation at one (multiple) remote electrically isolated reservoir(s)^8,9^. Non-local coupling between electrical and thermal parameters provides a number of distinct benefits over their local counterparts, which encompass isolation of the remote target reservoir from current flow induced Joule heating, the provision of independent engineering and manipulation of electrical and lattice thermal conductance, etc. Recently proposals towards non-local thermometry via thermoelectric voltage measurement in a capacitively coupled dual quantum dot set-up^10^ and current measurement in a point contact set-up^11^ have been put forward in literature. In such systems, the temperature of a remote target reservoir may be assessed via measurement of thermoelectric voltage or current between two terminals that are electrically isolated from the target reservoir^10,11^. In addition, a lot of effort has been directed towards theoretical and experimental demonstration of “sub-Kelvin range” thermometers^12,13^.

In this paper, we first argue that non-local thermoelectric voltage based sensitivity in the conventional dual dot set-up, proposed in Ref.^10^, is dependent on the average temperature of the measurement terminals, which might affect temperature assessment. Following this, we illustrate that non-local current-based thermometry offers an alternative and robust approach where the sensitivity remains unaffected by the average temperature of the measurement terminals. Although current based thermometry in the dual dot set-up^10^ offers an attractive alternative, the optimal performance of such a set-up demands a sharp step-like transition in the system-to-reservoir coupling, which is hardly achievable in reality. Hence, we propose a triple quantum dot based non-local thermometer that can perform optimally, while circumventing the demand for any energy resolved change in the system-to-reservoir coupling. The triple dot thermometer, proposed in this paper, is asymmetric and prone to non-local thermoelectric action due to the possibility of a difference in reservoir temperatures^1^. We, however, show that its thermometry remains practically unaffected by non-local thermoelectric action in the regime of high bias voltage. The performance and operation regime of the triple dot thermometer is investigated and compared with the conventional dual dot set-up to demonstrate that the triple dot thermometer offers enhanced temperature sensitivity along with a reasonable efficiency, while bypassing the demand for unrealistic step-like system-to-reservoir coupling and providing robustness against fabrication induced variability in Coulomb coupling. It is also demonstrated that the heat-extraction from the remote (non-local) target reservoir^7,9^ in the triple dot set-up can be substantially suppressed, without affecting the system sensitivity, by tuning the dot to remote reservoir coupling. Thus the triple dot thermometer hosts a multitude of advantages, making it suitable for its realization and deployment in practical applications.

**Figure 1:**
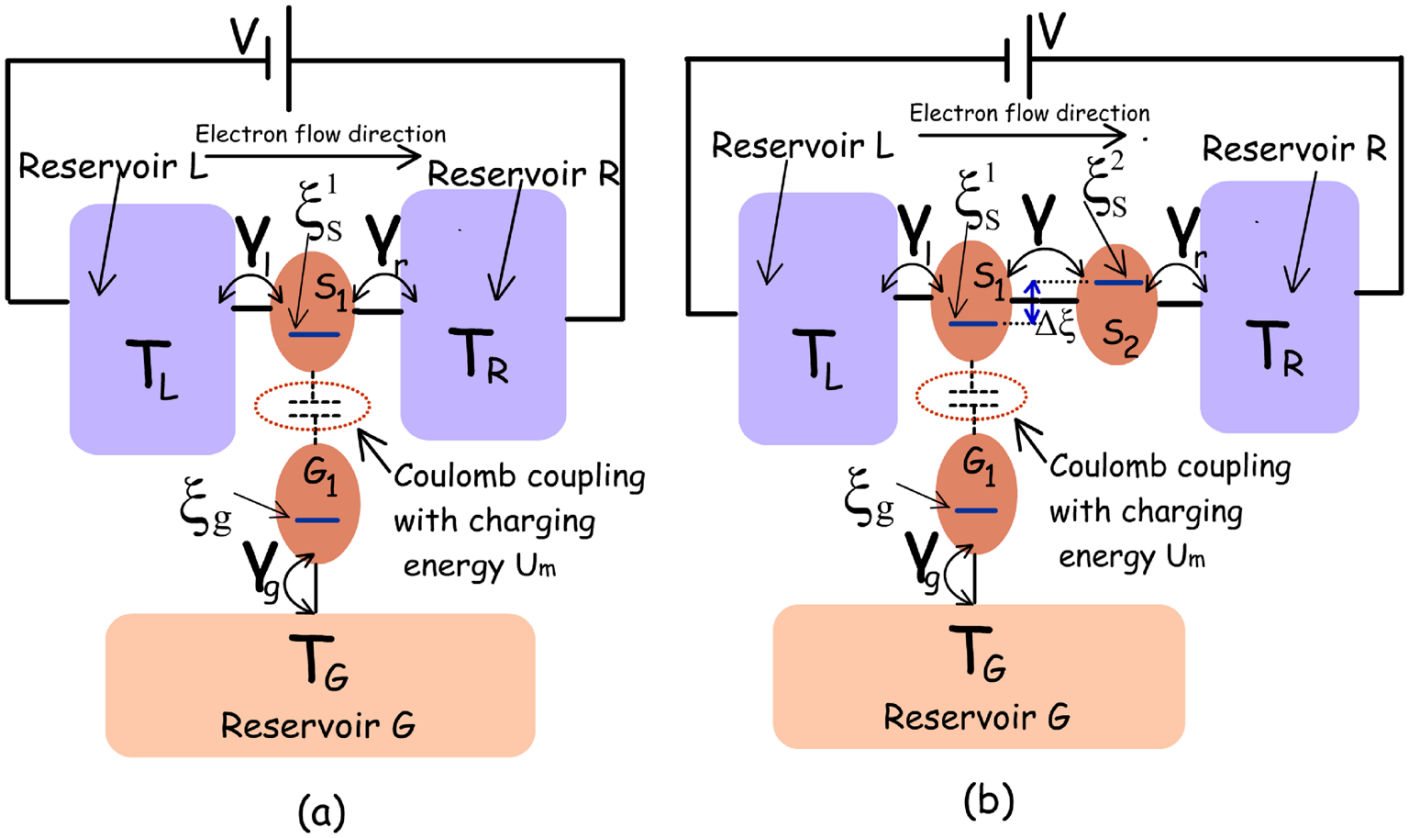
Schematic of the dual dot and triple dot thermometer (**a**) Schematic diagram of the dual dot thermometer based on Coulomb-coupled systems^10^. This thermometer set-up is based on a simpler thermodynamic engine proposed by Sánchez et al.^7^ and consists of two Coulomb-coupled quantum dots $S_1$ and $G_1$. $S_1$ is electrically connected to the reservoirs *L* and *R* and provides the path for current flow. $G_1$ on the other hand, is electrically connected to the remote reservoir *G* whose temperature is to be accessed. To investigate the optimal performance of the dual dot thermometer, we choose $\gamma _L(\xi )=\gamma _c \theta (\xi _s^1+\delta \xi -\xi )$, $\gamma _R(\xi )=\gamma _c\theta (\xi -\xi _s^1-\delta \xi )$ and $\gamma _g(\xi )=\gamma _c$^7^ with $\gamma _c=10\upmu$eV. Here, $\xi$ is the free variable denoting energy, $\theta$ is the Heaviside step function and $\delta \xi$ is a mathematical parameter that fixes the exact energy at which the transition in $\gamma _L(\xi )$ and $\gamma _R(\xi )$ occurs. For the particular arrangement discussed in this Refs.^7,14^ and in this paper, $\delta \xi <U_m$. (**b**) Schematic diagram of the proposed triple dot electrical thermometer. The entire system consists of the dots $S_1$, $S_2$ and $G_1$, which are electrically coupled to reservoirs *L*, *R*, and *G* respectively. $S_1$ and $G_1$ are capacitively coupled to each other (with Coulomb-coupling energy $U_m$). The ground state energy levels of the three dots $S_1$, $S_2$ and $G_1$ are denoted by $\xi _s^1$, $\xi _s^2$ and $\xi _g$ respectively. $S_1$ and $S_2$ share a staircase ground state configuration with energy difference $\Delta \xi$, such that $\xi _s^2=\xi _s^1+\Delta \xi$. To assess the optimal performance of the triple dot thermometer, we choose $\Delta \xi =U_m$ (see supplementary information) and $\gamma _L(\xi )=\gamma _r(\xi )=\gamma _g(\xi )=\gamma _c$, with $\gamma _c=10\upmu$eV.

## Results

In this section, we investigate non-local open-circuit voltage and current based thermometry in the dual dot set-up. Proceeding further, we propose a triple dot design that demonstrates a superior sensitivity while circumventing the demand for any change in the system-to-reservoir coupling. In addition, the triple dot thermometer also demonstrates robustness against fabrication induced variability in Coulomb coupling. The performance and operation regime in case of current based sensitivity for both the dual dot and the triple dot thermometers were investigated and compared. The last part of this section investigates the thermometry induced refrigeration (heat-up) of the remote reservoir in the dual and triple dot set-up and also elaborates a strategy to reduce such undesired effect in case of the triple dot design.

The two types of non-local thermometers recently proposed in literature include (i) open-circuit voltage based thermometers^10^, and (ii) current based thermometers^11^. Both of these thermometers rely on Coulomb coupling. The parameter employed to gauge the thermometer performance should be related to the rate of change of an electrical variable with temperature and is termed as sensitivity. As such, sensitivity is defined as the rate of change in (i) open-circuit voltage with temperature $\left( \frac{dV_o}{dT_G}\right)$ for voltage based thermometry and, (ii) current with temperature $\left( \chi = \frac{dI}{dT_G}\right)$ for current based thermometry. Here, $T_G$ is the remote target reservoir temperature to be assessed. When it comes to current based thermometry, a second parameter of importance, related to the efficiency, may be defined as the sensitivity per unit power dissipation, which weterm as the performance coefficient. Thus, performance coefficient is given by:

1 $$\begin{aligned} Performance-coefficient=\frac{\chi }{P}, \end{aligned}$$

where $P=V\times I$ is the power dissipated across the set-up. In the above equation, *I* indicates the current flowing through the thermometer on application of bias voltage *V*. It should be noted that the performance coefficient is a parameter to gauge the sensitivity with respect to power dissipation and is not a true efficiency parameter in sense of energy conversion.

### Thermometry in the dual dot set-up

The dual dot thermometer, schematically demonstrated in Fig. 1a, is based on the non-local thermodynamic engine originally conceived by Sánchez et al.^7^. It consists of two quantum dots $S_1$ and $G_1$. The dot $S_1$ is electrically tunnel coupled to reservoirs *L* and *R*, while $G_1$ is electrically coupled to the reservoir *G*. Here, *G* is the target reservoir whose temperature is to be assessed. The temperature of the reservoirs *L*,  *R* and *G* are symbolized as $T_L,~T_R$ and $T_G$ respectively. The dots $S_1$ and $G_1$ are capacitively coupled with Coulomb coupling energy $U_m$, which permits exchange of electrostatic energy between the dots $S_1$ and $G_1$ while prohibiting any flow of electrons between them, resulting in *zero* net electronic current out of (into) the reservoir *G*. Thus the reservoir *G* is electrically isolated from the current flow path. The ground state energy levels of the dots $S_1$ and $G_1$ are indicated by $\xi _s^1$ and $\xi _g$ respectively. It was demonstrated in Refs.^7,10^ that optimal operation of the dual-dot based set-up as heat engine and thermometer demands an asymmetric step-like system-to-reservoir coupling. Hence, to investigate the optimal performance of the dual dot thermometer, we choose $\gamma _l(\xi )=\gamma _c \theta (\xi _s^1+\delta \xi -\xi )$ and $\gamma _r(\xi )=\gamma _c\theta (\xi -\xi _s^1-\delta \xi )$^7^ with $\gamma _c=10\upmu$eV and $\delta \xi$ is a fixed number having the dimension of energy with $\delta \xi <U_m$ as already discussed in Ref.^1^. Here, $\theta$ and $\xi$ respectively are the Heaviside step function and the free-variable denoting energy. In addition, we choose $\gamma _g=\gamma _c$. Such order of coupling parameter correspond to realistic experimental values in Ref.^15^, where the system-to-reservoir coupling was evaluated, from experimental data, to lie in the range of $20\sim 50\upmu$eV. In addition, such order of the coupling parameters also indicate weak coupling and limit the electronic transport in the sequential tunneling regime where the impact of cotunneling and higher-order tunneling processes can be neglected. It should be noted that the coupling parameters $\gamma _{l(r)}$ are taken to be Heaviside step functions emulate the fact that electron can enter/exit from reservoir *L* to the dot $S_1$ through the energy $\xi _1$, but not through the level $\xi _1 + U_m$. This calls for using the function $\gamma _l(\xi )=\gamma _c \theta (\xi _s^1+\delta \xi -\xi )$, where $\delta \xi <U_m$, such that when $\xi =\xi _1$ then $\gamma _l=\gamma _c$. On the other hand when $\xi =\xi +U_m$, then $\gamma _l=0$. Similarly, it is required that the electron can enter/exit to the dot $S_1$ from the reservoir *R* through the energy level $\xi +U_m$, but not through the level $\xi _1$. This calls for using the function $\gamma _r(\xi )=\gamma _c\theta (\xi -\xi _s^1-\delta \xi )$, such that when $\xi =\xi _1$ then $\gamma _r=0$. On the other hand when $\xi =\xi _1 + U_m$, then $\gamma _r=\gamma _c$. Unless stated, the temperature of the reservoirs *L* and *R* are assumed to be $T_{L(R)}=300$mK. To assess the performance of the thermometer, we follow the approach as well as the quantum master equations employed in Refs.^7,9^, where the probability of occupancy of the considered multi-electron states were evaluated via well established quantum master equations (QME) to finally calculate the charge and heat currents through the system (See supplementary section for other details). On calculation of the charge and heat current, the different thermometry parameters, like sensitivity and performance-coefficient, may be calculated by using the formulas given in the previous paragraph.

**Figure 2:**
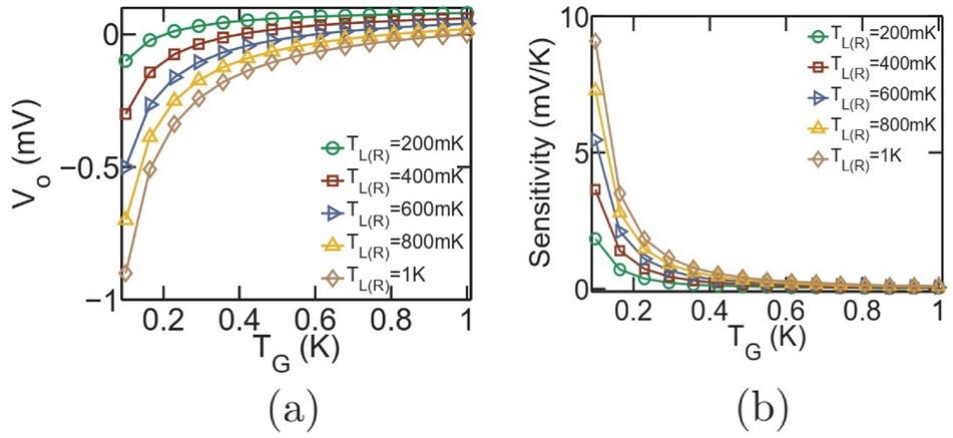
Voltage based thermometry in the dual-dot set-up depicted in Fig. 1a. Variation in (**a**) Open-circuit voltage (**b**) temperature sensitivity $\left( \frac{dV_o}{dT_G}\right)$ with $T_G$ for different values of $T_{L(R)}$. For the above set of plots, the value of Coulomb coupling energy is chosen as $U_m=100\upmu$eV and the ground states are pinned at the equilibrium Fermi energy, that is, $\xi _s^1=\xi _g=\mu _0$. The open-circuit voltage as well as temperature sensitivity $\left( \frac{dV_o}{dT_G}\right)$ in the set-up under consideration is dependent on $T_{L(R)}$.

**Figure 3:**
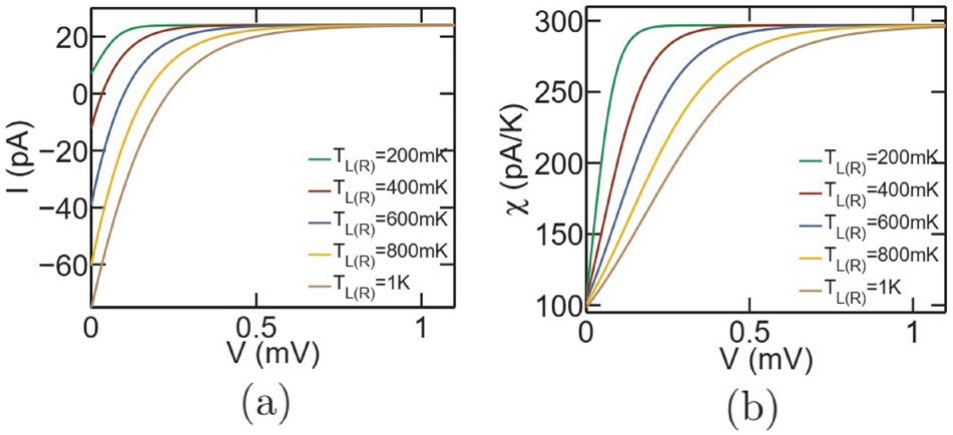
Current based thermometry in the dual-dot set-up depicted in Fig. 1a. Variation in (**a**) total current (**b**) temperature sensitivity $\left( \chi =\frac{dI}{dT_G}\right)$ with applied bias *V* for different values of $T_{L(R)}$. For the above set of plots, the parameters employed are $U_m=100\upmu$eV and $T_G=300$mK, while the ground states of $S_1$ and $G_1$ are pinned at the equilibrium Fermi energy, that is $\xi _s^1=\xi _g=\mu _0$. Given sufficiently high bias voltage *V*, the total current as well as temperature sensitivity $\chi =\left( \frac{dI}{dT_G}\right)$ saturate to the same value for different $T_{L(R)}$.

#### Voltage-based thermometry

In case of non-local thermoelectric voltage based thermometry, the applied bias *V* in Fig. 1a is replaced by open circuit and the voltage between the terminals *L* and *R* is measured. Such open circuit voltage based thermometry for the considered dual dot set-up was analyzed earlier in detail by Zhang et al.^10^. We plot, in Fig. 2, the variation in open-circuit voltage ($V_o$) and temperature sensitivity $\left( \frac{dV_o}{dT_G}\right)$ for different values of $T_{L(R)}$ at $U_m=100\upmu$eV. It is evident that the open-circuit voltage as well as sensitivity $\left( \frac{dV_o}{dT_G}\right)$ in such a set-up is dependent on $T_{L(R)}$, which makes it non-robust against fluctuations in the measurement terminal temperature. The variation in open-circuit voltage and sensitivity with $T_{L(R)}$ results from the fact that non-local thermoelectric voltage developed in such set-ups is dependent on $\Delta T =T_{L(R)}-T_G$. Due to the variation in sensitivity and open-circuit voltage with measurement terminal temperature, this strategy is unsuitable for deployment in practical applications. Hence, we will not discuss this strategy further.

**Figure 4:**
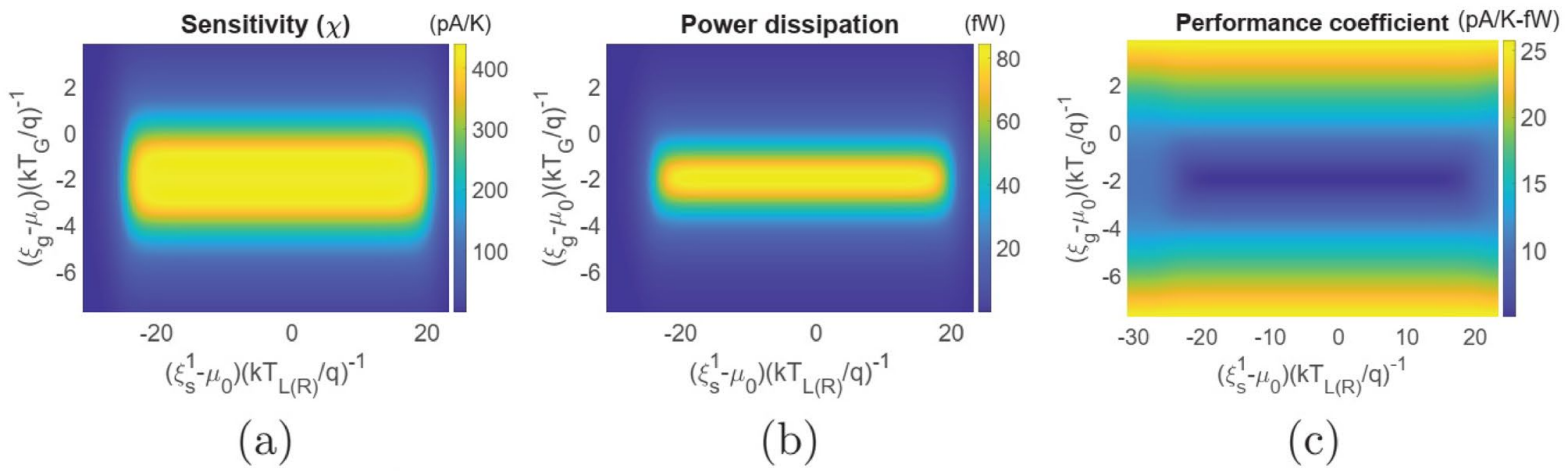
Regime of operation of the dual dot set-up in terms of the ground state energy positions relative to the equilibrium Fermi energy $\mu _0$. Colour plot demonstrating the variation in (**a**) sensitivity ($\chi$) (**b**) power dissipation and (**c**) performance coefficient with variation in the ground state positions $\xi _s^1$ and $\xi _g$. The parameters used for simulation are $U_m=100\upmu$eV, $\gamma _c=10\upmu$eV, $V=1.1$mV and $T_{L(R)}=T_G=300$mK.

#### Current-based thermometry

To ensure robustness in such a set-up against fluctuation and variation in measurement terminal temperature and voltage, current based thermometry offers an alternative method. In this case, a bias voltage *V* is applied between the reservoirs *L* and *R* and temperature of the reservoir *G* can be assessed via the current measurement. As stated before, temperature sensitivity in this case is defined as

2 $$\begin{aligned} \chi =\frac{dI}{dT_G}, \end{aligned}$$

where *I* is the electronic current flowing between the reservoirs *L* and *R*. Figure 3 demonstrates the variation in electronic current *I* and temperature sensitivity $\chi =\left( \frac{dI}{dT_G}\right)$ for different values of $T_{L(R)}$ at $U_m=100\upmu$eV. It should be noted that the set-up is affected by non-local thermoelectric action in the regime of low bias, which is evident from different magnitudes of current at distinct values of $T_{L(R)}$. However, for sufficiently high bias voltage, the electronic current as well as the sensitivity $\chi =\left( \frac{dI}{dT_G}\right)$ saturate to a finite limit for different values of $T_{L(R)}$. Thus, in the regime of high bias, current based thermometry in the set-up under consideration is robust against thermoelectric effect, fluctuations in the bias voltage and variation in measurement terminal temperature $T_{L(R)}$. Figure 4 demonstrates the regime of operation of the set-up under consideration with respect to the ground state energy positions for $U_m=100\upmu$eV, $V=1.1$mV and $T_{L(R)}=T_G=300$mK. Such values of the applied bias drive the thermometer in the regime of maximum saturation sensitivity. In particular, Fig. 4a demonstrates the variation in sensitivity ($\chi$) with ground state positions $\xi _s^1$ and $\xi _g$ relative to the equilibrium Fermi level $\mu _0$. We note that the optimal sensitivity is obtained when $\xi _g$ lies within the range of a few $kT_G$ below the equilibrium Fermi energy $\mu _0$. This is because, the flow of an electron from reservoir *L* to *R* demands the entry of an electron in dot $G_1$ at energy $\xi _g+U_m$ and subsequently exit of the electron from $G_1$ into reservoir *G* at an energy $\xi _g$^7,9^. To understand this, let us consider the complete cycle that transfers an electron from reservoir *L* to *R* in the dual dot set-up: $|0,0\rangle \rightarrow |1,0\rangle \rightarrow |1,1\rangle \rightarrow |0,1\rangle \rightarrow |0,0\rangle$. Here, $|n_{S_1},n_{G_1}\rangle$ denote a state of the entire set-up and $n_{S_1(G_1)}$ indicates the number of electrons in the ground state of the dot $S_1(G_1)$, with $n_{S_1},~n_{G_1} \in (0,1)$. In this cycle, an electron tunnels into the dot $S_1$ from reservoir *L* at energy $\xi _s^1$. Next, an electron tunnels into the dot $G_1$ from reservoir *G* at energy $\xi _g+U_m$. In the following step, the electron in $S_1$ tunnels out into the reservoir *R* at energy $\xi _s^1+U_m$. The system returns to the vacuum state, that is $|0,0\rangle$ when the electron in $G_1$ tunnels out into reservoir *G* at energy $\xi _g$. Thus, the sensitivity becomes optimal in the regime around the maximum value of the factor $\frac{d}{dT_G}\left[ f\left( \frac{\xi _g+U_m-\mu _0}{kT_G}\right) \left\{ 1-f\left( \frac{\xi _g-\mu _0}{kT_G}\right) \right\} \right]$, which occurs when $\xi _g$ is a few $kT_G$ below the equilibrium Fermi energy $\mu _0$. Similarly, the power dissipation, shown in Fig. 4b, is high when $\xi _g$ lies within the range of a few $kT_G$ below the equilibrium Fermi energy $\mu _0$ due to high current flow. Interestingly, by comparing Fig. 4a and b, we find regimes where the sensitivity is high at a relatively lower power dissipation. The performance coefficient (shown in Fig. 4c), on the other hand, is low in the regime of high sensitivity and increases as $\xi _g$ deviates from the equilibrium Fermi energy beyond a few $kT_G$. This can be explained as follows. In the regime of high sensitivity, the current flow is high. Due to limited current carrying capacity of the dual dot set-up, the rate of fractional increase in current flow with $T_G$, that is $\left( \frac{1}{I}\frac{dI}{dT_G}\right)$, is lower in the regime of high current flow. Hence, although the sensitivity is high, the rate of fractional increase in current flow with temperature, and hence the sensitivity per unit power dissipation is lower. This gives rise to low performance coefficient. On the other hand, in the regime of low sensitivity, the current flow is lower (evident from the lower power dissipation). Thus, the rate of fractional increase in current flow with $T_G$, that is $\left( \frac{1}{I}\frac{dI}{dT_G}\right)$, is higher in this regime. This gives rise to high performance coefficient in the regime of low sensitivity. From Fig. 4a–c, we also note that the sensitivity, power dissipation and performance coefficient is fairly constant over a wide range of $\xi _s^1$. Although not shown here, this range depends on and increases (decreases) with the increase (decrease) in the applied bias voltage.

**Figure 5:**
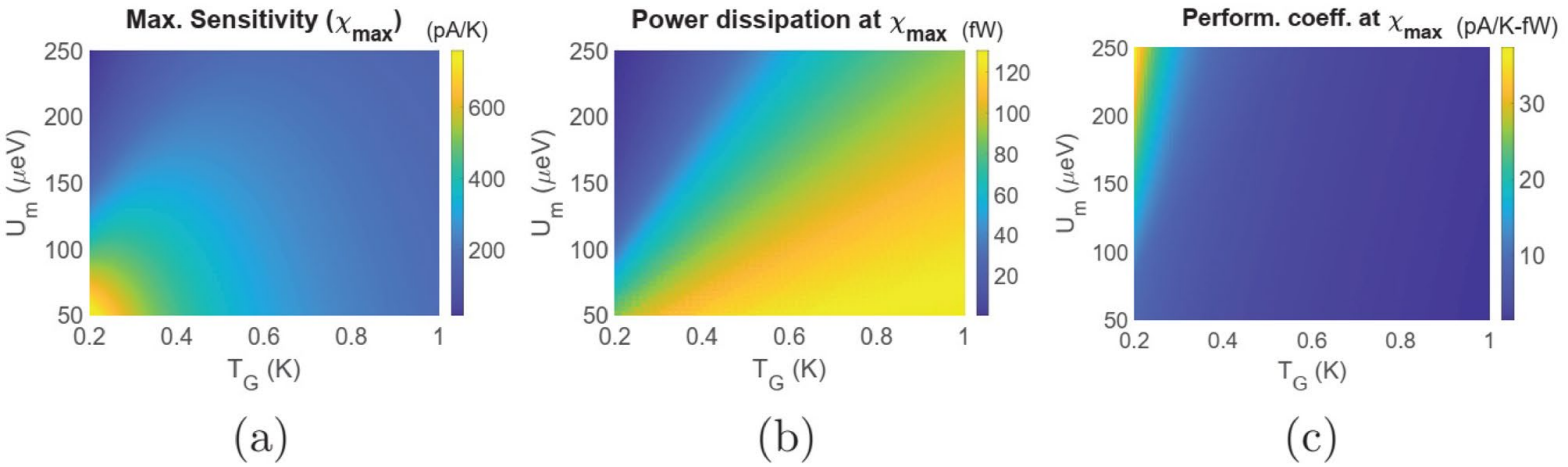
Maximum sensitivity and parameters at maximum sensitivity for the dual dot thermometer. Colour plot demonstrating the variation in (**a**) maximum sensitivity ($\chi _{max}$) (**b**) power dissipation at maximum sensitivity and (**c**) performance coefficient at maximum sensitivity with variation in the Coulomb coupling energy ($U_m$) and target reservoir temperature ($T_G$). The parameters used for simulation are $T_{L(R)}=300$mK, $\gamma _c=10\upmu$eV and $V=1.1$mV.

We demonstrate in Fig. 5, the variation in maximum sensitivity ($\chi _{max}$), as well as, power dissipation and performance coefficient at the maximum sensitivity with variation in the Coulomb coupling energy ($U_m$) and $T_G$ respectively. To calculate the maximum sensitivity and related parameters at the maximum sensitivity, the ground states are tuned to optimal positions with respect to the equilibrium Fermi energy ($\mu _0$). We note that the maximum sensitivity, shown in Fig. 5a, is relatively higher in the regime of low Coulomb coupling energy $U_m$ and decreases with $U_m$. This is because the maximum value of $\frac{d}{dT_G}\left[ f\left( \frac{\xi _g+U_m-\mu _0}{kT_G}\right) \left\{ 1-f\left( \frac{\xi _g-\mu _0}{kT_G}\right) \right\} \right]$ decreases with increase in $U_m$. Moreover, we also note that the sensitivity changes non-monotonically with $T_G$. Coming to the aspect of power dissipation, we note that the dissipated power at the maximum sensitivity decreases monotonically with $U_m$. This, again, is due to decrease in the optimal value of the product $f\left( \frac{\xi _g+U_m-\mu _0}{kT_G}\right) \left\{ 1-f\left( \frac{\xi _g-\mu _0}{kT_G}\right) \right\}$ with $U_m$, which results in decrease in the current flow and, hence power dissipation. In addition, the power dissipation also increases with $T_G$ for the same reason of increase in current due to increase in the product $f\left( \frac{\xi _g+U_m-\mu _0}{kT_G}\right) \left\{ 1-f\left( \frac{\xi _g-\mu _0}{kT_G}\right) \right\}$ with $T_G$. The performance coefficient at the maximum sensitivity, as noted from Fig. 5c, is maximum in the regime of low temperature and high Coulomb coupling energy $U_m$, rendering this set-up suitable for applications in the “sub-Kelvin” temperature regime.

### Thermometry in triple-dot set-up

#### Proposed set-up configuration

The dual dot thermometer, discussed above, suffers in a few crucial points, which include (i) demand for unrealistic step-like system-to-reservoir coupling (ii) thermometry induced refrigeration of the remote target reservoir (discussed later), and (iii) change in maximum sensitivity due to fabrication induced variability in Coulomb coupling energy $U_m$ (Fig. 5a). The triple dot thermometer, discussed below, alleviates these issues and hence is suitable for deployment in practical applications. The triple dot thermometer, proposed in this paper, is schematically demonstrated in Fig. 1b and consists of three dots $S_1,~S_2$ and $G_1$ which are electrically coupled to the reservoirs *L*, *R* and *G* respectively. Compared to the dual-dot design, the triple dot set-up features an extra quantum dot $S_2$ between $S_1$ and reservoir *R*. Coming to the ground state configuration and other features of the system, $S_1$ and $S_2$ are tunnel coupled to each other, while $G_1$ is capacitively coupled to $S_1$. The ground states of $S_1$ and $S_2$ form a stair-case configuration with $\xi _s^2= \xi _s^1+\Delta \xi$. Any electronic tunneling between the dots $S_1$ and $G_1$ is suppressed via suitable fabrication techniques^16–20^. Energy exchange between $S_1$ and $G_1$ is, however, feasible via Coulomb coupling^16–20^. In the optimal dual-dot thermometer discussed above, an asymmetric step-like system-to-reservoir coupling is required for optimal operation. In the proposed triple-dot thermometer, the asymmetric system-to-reservoir coupling is bypassed by choosing an energy difference between the ground states of $S_1$ and $S_2$ which makes the system asymmetric with respect to the reservoir *L* and *R*. Another equivalent triple-dot set-up, based on Coulomb coupled systems, that can be employed for efficient non-local thermometry is demonstrated and discussed briefly in the Supplementary material. Coming to the realistic fabrication possibility of such a system, due to the recent advancement in solid-state nano-fabrication technology, triple and quadruple quantum dot systems with and without Coulomb coupling have already been realized experimentally^21–23^. In addition, it has been experimentally demonstrated that quantum dots that are far from each other in space, may be bridged to obtain strong Coulomb coupling, along with excellent thermal isolation between the reservoirs which may be at different temperatures^16–20^. Also, the bridge may be fabricated between two specific quantum dots to drastically enhance their mutual Coulomb coupling, without affecting the electrostatic energy of the other quantum dots in the system^16–20^.

**Figure 6:**
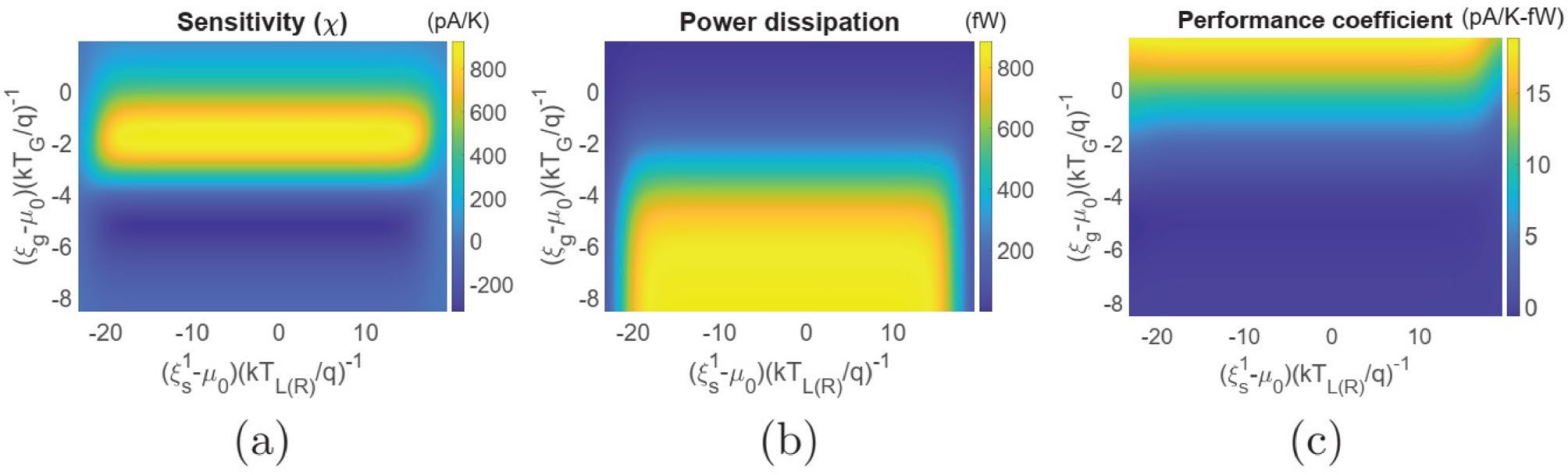
Regime of operation of the proposed triple dot thermometer in terms of the ground state energy positions relative to the equilibrium Fermi energy $\mu _0$. Colour plot demonstrating the variation in (**a**) sensitivity ($\chi$) (**b**) power dissipation and (**c**) performance coefficient with variation in the ground state positions $\xi _s^1$ and $\xi _g$. The parameters used for simulation are $U_m=100\upmu$eV, $\gamma _c=10\upmu$eV, $V=1.1$mV and $T_{L(R)}=T_G=300$mK.

**Figure 7:**
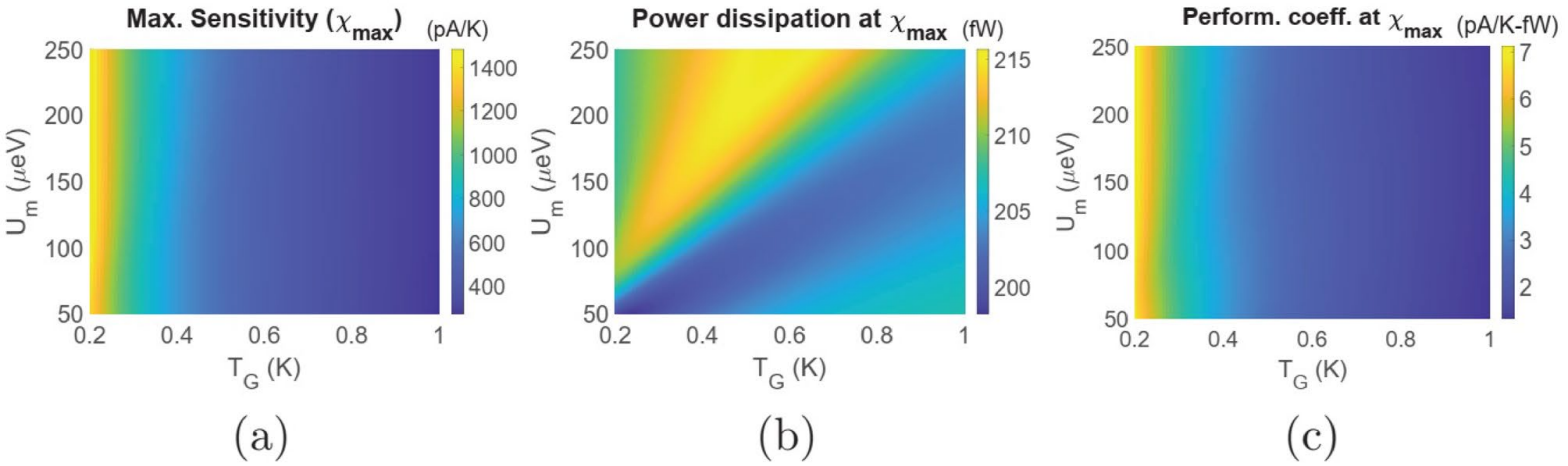
Maximum sensitivity and parameters at maximum sensitivity for the triple dot thermometer. Colour plot demonstrating the variation in (**a**) maximum sensitivity ($\chi _{max}$) (**b**) power dissipation at maximum sensitivity and (**c**) performance coefficient at maximum sensitivity with variation in the Coulomb coupling energy ($U_m$) and target reservoir temperature ($T_G$). The parameters used for simulation are $T_{L(R)}=300$mK, $\gamma _l(\xi )=\gamma _r(\xi )=\gamma _g(\xi )=\gamma (\xi )=\gamma _c=10\upmu$eV and $V=1.1$mV.

#### Operation regime and performance investigation

For investigating the triple dot set-up, we choose the system-to-reservoir coupling as $\gamma _l(\xi )=\gamma _r(\xi )=\gamma _g (\xi )=\gamma _c$, with $\gamma _c=10\upmu$eV. In addition, we also choose the interdot coupling to be $\gamma (\xi )=10\upmu$eV. As stated earlier, such values of coupling parameters lie within experimentally feasible range^15^. Figure 6 demonstrates the regime of operation of the proposed triple dot thermometer. In particular, Fig. 6a depicts the sensitivity as a function of the ground state positions. We note that the sensitivity increases as $\xi _g$ gradually goes below the Fermi energy, with the maximum sensitivity occurring when $\xi _g-\mu _0 \approx -1.5kT_G$. As $\xi _g$ goes further below the Fermi energy, the sensitivity becomes negative. This occurs when an increase in temperature decreases the probability of occupancy of both the ground state $\xi _g$ and the Coulomb blocked state $\xi _g+U_m$, that is when $\xi _g+U_m-\mu _0<0$. Despite the fact that this set-up offers the provision to implement a positively sensitive as well as a negatively sensitive thermometer, it should be noted from Fig. 6b that the power dissipation is very high in the negatively sensitive regime. This is due to the fact that when $\xi _g+U_m-\mu _0<0$, the occupancy probability of $G_1$ is very high, which causes a high drive current between reservoirs *L* and *R*. The power dissipation in the regime of positive sensitivity is lower, resulting in a higher performance coefficient, as noted from Fig. 6c. Also, the power dissipation and performance coefficient respectively decreases and increases as $\xi _s^1$ gradually approaches and finally moves above the equilibrium Fermi-energy. This is because as $\xi _g$ gradually approaches and goes above the Fermi energy, the probability of occupancy of $\xi _g$ becomes lower, blocking the current flow through the system. Due to the same reason as stated for the dual dot set-up, a lower current flow through the system leads to a higher fractional increase in current with the remote reservoir temperature $T_G$, leading to a higher performance coefficient. We also note from Fig. 6a–c that the sensitivity, power dissipation and performance coefficient remains almost constant for a wide range of $\xi _s^1$. As discussed before, this range depends on and increases (decreases) with increase (decrease) in applied bias voltage.

**Figure 8:**
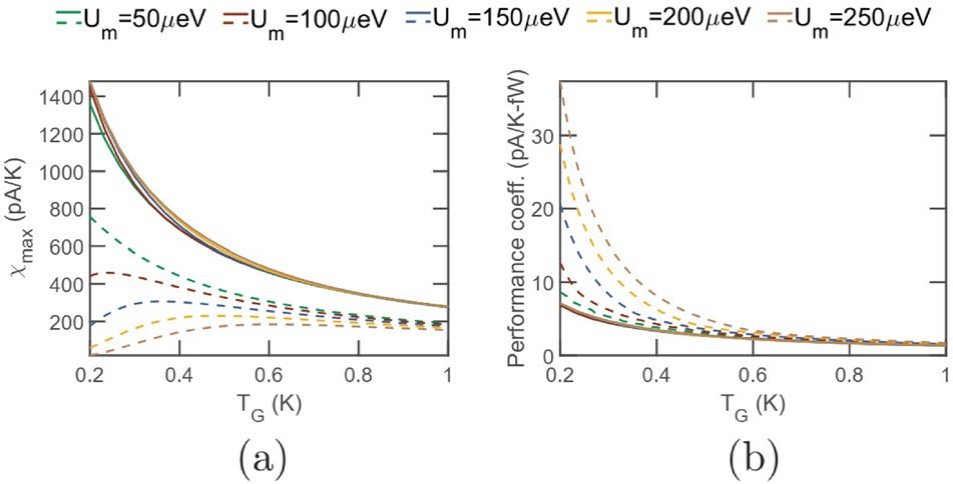
Performance comparison between the dual dot and the triple dot thermometer. Variation in (**a**) maximum sensitivity ($\chi _{max}$) and (**b**) Performance-coefficient at the maximum sensitivity with $T_G$ for different values of Coulomb coupling energy $U_m$. The solid and the dashed line represent the performance parameters of the triple dot and dual dot thermometers respectively. The system parameters used for simulation are $T_{L(R)}=300$mK, and $V=1.1$mV. For the dual dot thermometer, the different system to reservoir coupling are chosen to be $\gamma _l(\xi )=\gamma _c \theta (\xi _s^1+\delta \xi -\xi )$, $\gamma _r(\xi )=\gamma _c\theta (\xi -\xi _s^1-\delta \xi )$ and $\gamma _g=\gamma _c$. For the triple dot thermometer, the system to reservoir, as well as the interdot coupling are chosen to be $\gamma _l(\xi )=\gamma _r(\xi )=\gamma _g(\xi )=\gamma (\xi )=\gamma _c=10\upmu$eV. In both the dual dot and the triple dot thermometer, $gamma_c=10\upmu$eV.

Figure 7 demonstrates the maximum sensitivity ($\chi _{max}$) as well as the power dissipation and performance coefficient at the maximum sensitivity with variation in the Coulomb coupling energy $U_m$ and target reservoir temperature $T_G$. Just as before, to calculate the maximum sensitivity and related parameters at the maximum sensitivity, the quantum dot ground states are tuned to their optimal positions. Figure 7a demonstrates the maximum sensitivity with variation in $U_m$ and $T_G$. An interesting thing to note is that the triple dot thermometer is fairly robust against variation in the Coulomb coupling energy $U_m$. This can be explained by the fact that current flow through the triple quantum dot set-up only demands the occupancy of the dot $G_1$ whose ground state can be tuned to optimum position for maximizing the sensitivity. Thus, optimal sensitivity can be achieved by placing $\xi _g$ around the energy $\xi$ at which the rate of change in ground state occupancy probability of $G_1$ is maximum with $T_G$. This condition is unlike the case of dual dot set-up where one has to maximize the factor $\frac{d}{dT_G}\left[ f\left( \frac{\xi _g+U_m-\mu _0}{kT_G}\right) \left\{ 1-f\left( \frac{\xi _g-\mu _0}{kT_G}\right) \right\} \right]$ for achieving the maximum sensitivity. We also note that, unlike the dual dot set-up, the maximum sensitivity in this case decreases monotonically with $T_G$. The power dissipation, as demonstrated in Fig. 7b, also remains almost constant and varies between 199fW and 216fW with variation in $U_m$ and $T_G$. This again is a result of the fact that current flow through the triple dot set-up only demands occupancy of the dot $G_1$ and thus the position of $\xi _g$ for maximum sensitivity induces a high current flow through the set-up. Due to almost constant power dissipation with variation in $U_m$ and $T_G$, the performance-coefficient also shows a similar trend as the sensitivity with $U_m$ and $T_G$, as noted in Fig. 7c. It is evident from Figs. 4, 5, 6 and 7 that the triple dot thermometer demonstrates an enhanced sensitivity, but lower performance coefficient compared to the dual dot thermometer. As such, it is important to compare their performance, which leads us to the next discussion.

### Performance comparison

To further shed light on the relative performance of the triple dot thermometer with respect to the dual dot thermometer, we plot in Fig. 8a and b the sensitivity and performance-coefficient respectively for the dual dot (dashed lines) and the triple dot (solid lines) thermometers respectively. As stated earlier, the triple dot thermometer demonstrates an enhanced sensitivity and offers significant advantage, particularly in the regime of high Coulomb coupling energy $U_m$. This is due to the fact that each electronic flow between reservoirs *L* and *R* in the dual dot set-up demands an electron entrance and exit from $G_1$ at energy $\xi _g+U_m$ and $\xi _g$ respectively. Thus, the probability of electronic flow is significantly reduced, particularly for high $U_m$. Electronic flow in the triple dot set-up on the other hand demands only occupancy of the dot $G_1$, which can be achieved by positioning the ground state $\xi _g$ appropriately with respect to the equilibrium Fermi energy. Thus, this system eliminates the dependence of sensitivity on $U_m$, making it fairly robust against fabrication induced variability in the Coulomb coupling energy. The performance coefficient of the triple dot set-up, on the other hand, is lower compared to the dual dot thermometer. This is due to high current flow in the triple dot thermometer and becomes particularly noticeable in the regime of high values of $U_m$, where the dual dot set-up hosts very less current flow and sensitivity but high performance coefficient. It should be noted that the performance coefficient offered by the triple dot thermometer is reasonable and approaches that of the dual dot set-up in the higher temperature regime.

**Figure 9:**
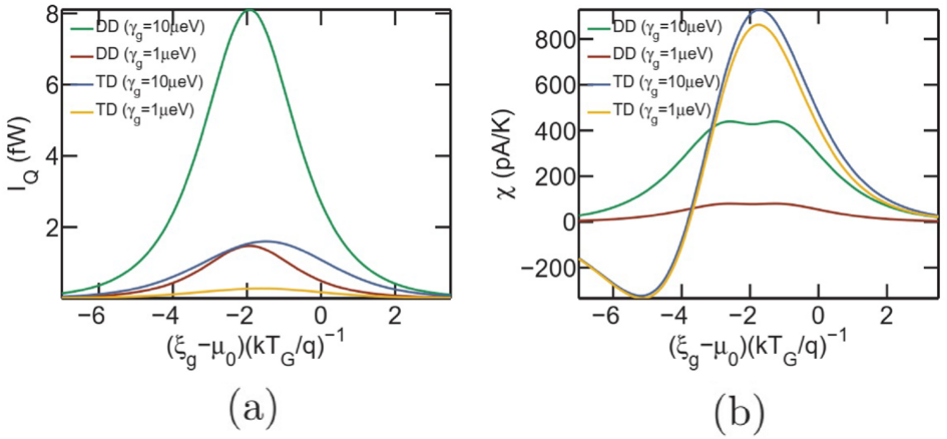
Analysis of thermometry induced refrigeration of the reservoir *G* for the dual-dot (DD) and triple dot (TD) set-up. Plot of (**a**) heat current ($I_Q$) extracted from the reservoir *G* and (**b**) sensitivity with variation in the ground state $\xi _g$. In case of the dual dot (DD) set-up, decreasing the system-to-reservoir coupling ($\gamma _g$) between *G* and $G_1$ decreases both the extracted heat current $I_Q$ and sensitivity $\chi$. However, for the triple dot set-up, decreasing $\gamma _g$ suppresses only the heat current $I_Q$, while keeping the sensitivity ($\chi$) almost unaltered. The parameters used for simulation are $U_m=100\upmu$eV, $\gamma _c=10\upmu$eV, $T_{L(R)}=T_G=300$mK and $\xi _s^1=\mu _0$.

### Thermometry induced refrigeration

It is well known that the transfer of each electron from reservoir *L* to *R*, in the dual dot set-up, demands extraction of a heat packet $U_m$ from reservoir *G*^7,9^. This means that increasing the system-to-reservoir coupling to achieve enhanced sensitivity would also result in extraction of more heat packets from reservoir *G*. Such a phenomena may result in unnecessary refrigeration or temperature drift of the reservoir *G* in an undesirable manner. Since, the number of heat packets extracted in this set-up is exactly equal to the number of electrons that flow between reservoir *L* and *R* ($I_Q=IU_m/q$), reducing $\gamma _g$ to suppress the refrigeration of reservoir *G* also results in the reduction of sensitivity. This is shown in Fig. 9a and b, where it is demonstrated that reduction in $\gamma _g$ for the dual dot (DD) set-up, by a factor of 10, results in suppression of both the maximum heat current ($I_Q$) from 8.1fW to 1.47fW and maximum sensitivity ($\chi$) from 440pA/K to 80pA/K. Thus, both the maximum heat current and maximum sensitivity decrease by a factor of approximately 5.5

**Figure 10:**
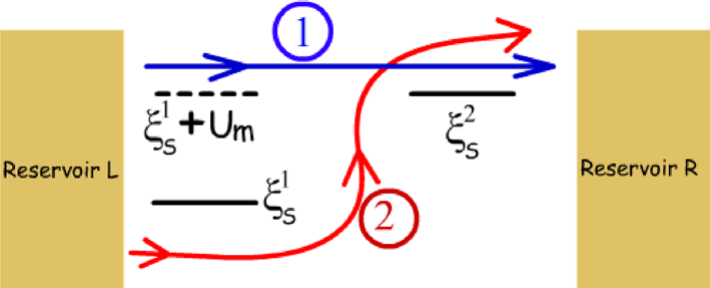
Schematic diagram depicting the two current components through the triple dot set-up. “Component 1” (directed blue arrow) flows without absorbing heat packets from the remote reservoir *G* and only depends on the occupancy probability of the ground state of $G_1$. “Component 2” (directed red line) flows by absorbing heat packets from the reservoir *G*, and results in extraction of heat from the same.

In this aspect of refrigeration of the target reservoir *G*, the proposed triple dot set-up, on the other hand, offers a significant edge over the dual dot set-up. It should be noted that an electron flow in the triple dot set-up does not always demand the extraction of a heat packet from the reservoir *G*. To understand this, the components of current flow in the triple dot set-up are demonstrated in Fig. 10. As noted from Fig. 10, “Component 1” flows directly from reservoir *L* to *R*, without absorbing heat packets from reservoir *G*. This component flows when the ground state of the dot $G_1$ is occupied. Hence, it depends mainly on the probability of occupancy of the dot $G_1$ and is not directly controlled by the parameter $\gamma _g$. “Component 2”, on the other hand, flows when the electron enters in the dot $S_1$ with unoccupied ground state of the dot $G_1$. In this case, the electronic flow occurs through the system as follows: (i) An electron enters the unoccupied dot $S_1$ at energy $\xi _s^1$. (ii) This is followed by another electron tunneling into the ground state of the dot $G_1$ at energy $\xi _g+U_m$. (iii) Next, the electron in $S_1$ tunnels out at energy $\xi _s^2=\xi _s^1+U_m$ into the dot $S_2$ and finally to the reservoir *R*. (iv) At the end of the cycle the electron in $G_1$ tunnels out into reservoir *G* at energy $\xi _g$. Hence, each electron in this component flows by absorbing heat packet of $U_m$ from reservoir *G* and depends on the rate at which electrons can enter and exit the dot $G_1$ at energy $\xi _g+U_m$ and $\xi _g$ respectively. Thus, this component depends on $\gamma _g$ and can be suppressed substantially by reducing $\gamma _g$. Thus, on decreasing $\gamma _g$, the magnitude of the heat current from reservoir *G* can be suppressed substantially.

As demonstrated in Fig. 9a and b, the triple dot setup extracts much lower heat current from the reservoir *G*, while offering an enhanced sensitivity. In addition, the heat current can be suppressed by a large amount without much impact on the sensitivity by decreasing $\gamma _g$. This is clearly demonstrated in Fig. 9a and b, where decreasing $\gamma _g$ by a factor of 10 in the triple dot (TD) set-up decreases the maximum extracted heat current from 1.6fW to 0.276fW (by a factor of almost 5.8), while keeping the sensitivity almost unchanged. Thus, a smart fabrication strategy in the triple dot set-up may be employed to prevent thermometry induced refrigeration and temperature drift of the remote target reservoir *G*.

## Discussion

To conclude, in this paper, we have proposed current based non-local thermometry as a robust and practical alternative to thermoelectric voltage based operation. Subsequently, we have investigated current based thermometry performance and regime of operation of the conventional dual dot set-up. Proceeding further, we have proposed a triple dot non-local thermometer which demonstrates a higher sensitivity while bypassing the need for unrealistic step-like system-to-reservoir coupling, in addition to providing robustness against fabrication induced variability in the Coulomb coupling energy. Furthermore, it was demonstrated that suitable fabrication strategy in the triple dot set-up aids in suppressing thermometry induced refrigeration (heat-up) and temperature drift in the remote target reservoir to a significant extent. Thus, the triple dot set-up hosts multitude of advantages that are necessary to deploy quantum non-local thermometers in practical applications. In this paper, we have mainly considered the limit of weak coupling which restricts electronic transport in the sequential tunneling regime and validates the use of quantum master equation for system analysis. It would, however, be interesting to investigate the impacts of cotunneling on the thermometer performance as the system is gradually tuned towards the strong coupling regime. In addition, an analysis on the impacts of electron-phonon interaction on the system performance would also constitute an interesting study. Other practical design strategies for non-local quantum thermometers is left for future investigation. Nevertheless, the triple dot design investigated in this paper can be employed to fabricate highly sensitive and robust non-local “sub-Kelvin” range thermometers.

## Method

The modeling is done using modified Liouville equation^24^ for open quantum systems in the weak coupling limit. The sets of modified Liouville equations were transformed to rate equations to solve the dynamics of the system. The simulations were done using MATLAB 2021a^25^ and Newton-Raphson iterative method was employed to solve the steady-state values of system state probabilities. The detailed formulation, as well as relevant derivations are given in the supplementary information.

## Supplementary Information

Supplementary Information.

## Data Availability

The authors declare that the main data supporting the findings of this study are available within the paper and its Supplementary Information file. The MATLAB codes are available from the authors upon request.
