# Supplementary material for: Non-local triple quantum dot thermometer based on Coulomb-coupled systems
Source: Sci Rep. 2022 Sep 23;12:15842. doi: 10.1038/s41598-022-19596-5 (PMC9508108; doi:10.1038/s41598-022-19596-5)
Supplement: Supplementary file 1 — Supplementary Information. [file 41598_2022_19596_MOESM1_ESM.pdf]

Non-local triple quantum dot thermometer based on Coulomb-coupled systems

Suraj G. Dhongade[1,+], Afreen A. Haque[1,+], Sayan Saha Roy[1], and Aniket Singha[1,*]

[1]Department of Electronics and Electrical Communication Engineering,, Indian Institute of Technology Kharagpur, Kharagpur-721302, India
[*]aniket@ece.iitkgp.ac.in
[+]these authors contributed equally to this work

ABSTRACT

This document presents the derivations of the quantum master equations (QME) for the triple dot system, that are used in the main manuscript in addition to highlighting the main assumptions for electronic transport in the dual dot set-up. It also describes another alternative triple dot system configuration for non-local thermometry.

1 Transport formulation in the dual-dot set-up

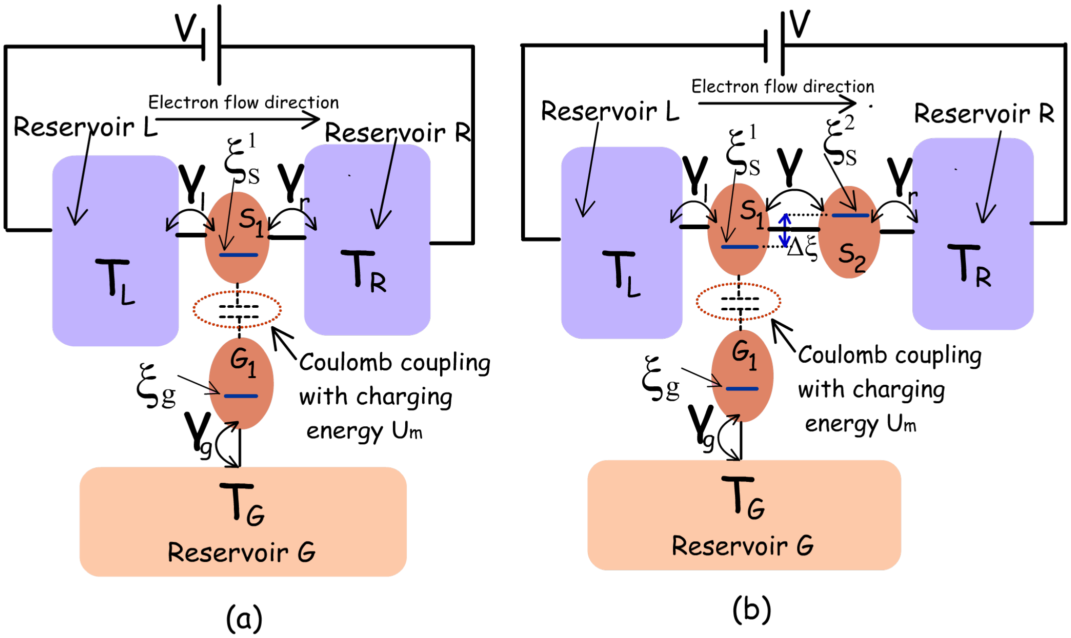

Supplementary Figure S1: Schematic of the dual dot and triple dot thermometer (a) Schematic diagram of the dual dot thermometer based on Coulomb-coupled systems[1]. This thermometer set-up is based on a simpler thermodynamic engine proposed by Sánchez *et. al.*[2] and consists of two Coulomb-coupled quantum dots S_1 and G_1. S_1 is electrically connected to the reservoirs L and R and provides the path for current flow. G_1 on the other hand, is electrically connected to the remote reservoir G whose temperature is to be accessed. To investigate the optimal performance of the dual dot thermometer, we choose $\gamma_L(\xi) = \gamma_c\theta(\xi_s^1 + \delta\xi - \xi)$, $\gamma_R(\xi) = \gamma_c\theta(\xi - \xi_s^1 - \delta\xi)$ and $\gamma_g(\xi) = \gamma_c$[2] with $\gamma_c = 10\mu$eV. Here, θ is the Heaviside step function and $\delta\xi < U_m$. (b) Schematic diagram of the proposed triple dot electrical thermometer. The entire system consists of the dots S_1, S_2 and G_1, which are electrically coupled to reservoirs L, R, and G respectively. S_1 and G_1 are capacitively coupled to each other (with Coulomb-coupling energy U_m). The ground state energy levels of the three dots S_1, S_2 and G_1 are denoted by ξ_s^1, ξ_s^2 and ξ_g respectively. S_1 and S_2 share a staircase ground state configuration with $\xi_s^2 = \xi_s^1 + \Delta\xi$. To assess the optimal performance of the triple dot thermometer, we choose $\Delta E = U_m$ and $\gamma_L(\xi) = \gamma_r(\xi) = \gamma_g(\xi) = \gamma_c$, with $\gamma_c = 10\mu$eV.

Due to mutual Coulomb coupling between S_1 and G_1 in the dual dot set-up demonstrated in Supplementary Figure S1(a), the change in electron number n_{S_1} (n_{G_1}) of the dot S_1 (G_1) influences the electrostatic energy of the dot G_1 (S_1). Under the assumption that the change in potential due to self-capacitance is much greater than the applied voltage V or the average thermal voltage kT/q, that is $q^2/C_{self} >> (qV, k_BT)$, the electron occupation probability or transfer rate via the Coulomb blocked energy level, due to self-capacitance can be neglected[2]. Thus, the system analysis can be approximated by considering four multi-electron levels by limiting the maximum number of electrons in the ground state of each quantum dot to 1. Denoting each state by the electron occupation number in the quantum dot ground state, a possible system state of interest may be represented as $|n_{S_1}, n_{G_1}\rangle = |n_{S_1}\rangle \otimes |n_{G_1}\rangle$, where $n_{S_1}, n_{G_1} \in (0,1)$, denote the number of electrons present in the ground-states of S_1 and G_1 respectively. The above assumptions validate the use of the quantum master equations (QME) employed in Ref.[2] for investigation of an equivalent set-up. It was demonstrated in Refs.[1,2] that optimal operation of the dual-dot based set-up as heat engine and thermometer demands an asymmetric step-like system-to-reservoir coupling. Hence, to investigate the optimal performance of the dual dot thermometer, we choose $\gamma_l(\xi) = \gamma_c\theta(\xi_s^1 + \delta\xi - \xi)$and $\gamma_r(\xi) = \gamma_c\theta(\xi - \xi_s^1 - \delta\xi)$[2] with $\gamma_c = 10\mu$eV and $\delta\xi < U_m$. Here, θ and ξ respectively are the Heaviside step function and the free-variable denoting energy. In addition, we choose $\gamma_g = \gamma_c$. Such order of coupling parameter correspond to realistic experimental values in Ref.[3], where the system-to-reservoir coupling was evaluated, from experimental data, to lie in the range of $20 \sim 50\mu$eV. In addition, such order of the coupling parameters also indicate weak coupling and limit the electronic transport in the sequential tunneling regime where the impact of cotunneling and higher-order tunneling processes can be neglected.

2 Derivation of quantum master equations (QME) for the triple dot thermometer

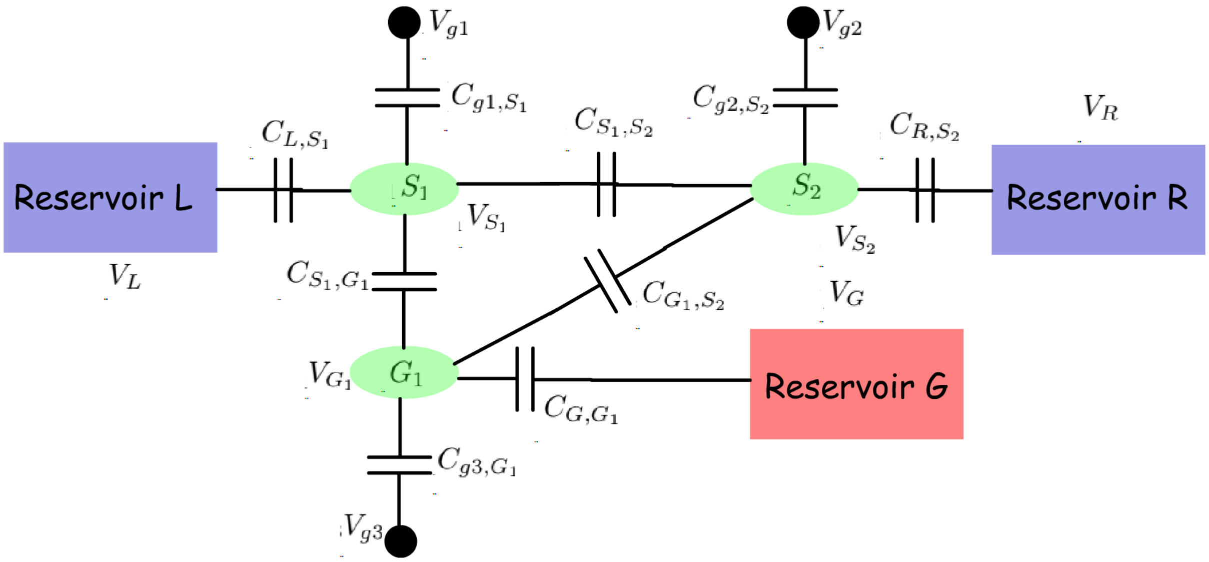

Supplementary Figure S2: Schematic diagram demonstrating electrostatic interaction of the system with the adjacent electrodes and other dots. The voltages V_{g1}, V_{g2}, V_{g3} are the voltages at the gate terminals of the dots S_1, S_2 and G_1 respectively.

In this section, I derive the quantum master equations (QME) for the proposed triple dot non-local thermometer, starting from the basic physics of Coulomb coupled systems. Supplementary Figure S2 depicts equivalent schematic model for electrostatic interaction of the quantum dots with the adjacent electrodes as well as the adjacent dots. V_{g1}, V_{g2}, V_{g3} are the voltages at the gate terminals of the dots S_1, S_2 and G_1 respectively. The other symbols in Supplementary Figure S2 are self-explanatory. The potentials of the dots S_1, S_2 and G_1 can be calculated in terms of the quantum dot charge and the potentials at the adjacent terminals as[4]:

$$V_{S_1} = \frac{Q_{S_1}}{C^{\Sigma}_{S_1}} + \frac{1}{C^{\Sigma}_{S_1}} \left\{ C_{g1,S_1} V_{g1} + C_{L,S_1} V_L + C_{S_1,S_2} V_{S_2} + C_{S_1,G_1} V_{G_1} \right\}$$
$$V_{G_1} = \frac{Q_{G_1}}{C^{\Sigma}_{G_1}} + \frac{1}{C^{\Sigma}_{G_1}} \left\{ C_{g3,G_1} V_{g3} + C_{G,G_1} V_G + C_{G_1,S_2} V_{S_2} + C_{S_1,G_1} V_{S_1} \right\}$$
$$V_{S_2} = \frac{Q_{S_2}}{C^{\Sigma}_{S_2}} + \frac{1}{C^{\Sigma}_{S_2}} \left\{ C_{g2,S_2} V_{g2} + C_{R,S_2} V_R + C_{G_1,S_2} V_{G_1} + C_{S_1,S_2} V_{S_1} \right\}, \tag{1}$$

where Q_x is the charge in dot x and the terms C^{Σ}_x is the total capacitance seen by the dot x with its adjacent environment.

$$C^{\Sigma}_{S_1} = C_{g1,S_1} + C_{S_1,G_1} + C_{L,S_1} + C_{S_1,S_2}$$
$$C^{\Sigma}_{S_2} = C_{g2,S_2} + C_{G_1,S_2} + C_{R,S_2} + C_{S_1,S_2}$$
$$C^{\Sigma}_{G_1} = C_{g3,G_1} + C_{S_1,G_1} + C_{G,G_1} + C_{G_1,S_2} \tag{2}$$

In general, each dot is coupled strongly with its corresponding gate terminal. Thus, from a practical purposes, the effective capacitance C_{L,S_1}, C_{G,G_1} and C_{R,S_2} between the quantum dots and the electrically-coupled electrodes can be neglected with respect to the gate coupling capacitances C_{g1,S_1}, C_{g2,S_2} and C_{g3,G_1}. In addition, the dots S_1 and G_1 are strongly coupled (intentionally) by suitable fabrication techniques[5–9]. In addition, we assume that electrostatic coupling between $S_1 - S_2$ and $G_1 - S_2$ are negligible. However, C_{S_1,G_1} is enhanced via appropriate fabrication techniques[5–9], such that $C_{S_1,G_1} >> (C_{S_1,S_2}, C_{G_1,S_2})$. Hence, for the following derivations, we neglect the capacitances C_{L,S_1}, C_{R,S_2}, C_{G,G_1}, C_{S_1,S_2}, C_{G_1,S_2}. Under all these considerations, the total system electrostatic energy can be given by[4]:

$$U_{tot} = \sum_{x \in (S_1,S_2,G_1)} \frac{Q_x^2}{2C^{\Sigma}_x} + \frac{Q_{S_1}}{C^{\Sigma}_{S_1}} \left\{ C_{g1,S_1} V_{g1} + C_{S_1,G_1} V_{G_1} \right\} + \frac{Q_{S_2}}{C^{\Sigma}_{S_2}} C_{g2,S_2} V_{g2} + \frac{Q_{G_1}}{C^{\Sigma}_{G_1}} \left\{ C_{g3,G_1} V_{g3} + C_{S_1,G_1} V_{S_1} \right\}, \tag{3}$$

where it is assumed that C_{L,S_1}, C_{R,S_2}, C_{G,G_1}, C_{S_1,S_2}, C_{G_1,S_2} is negligible compared to the other capacitances in the system. At $0K$, the system would equilibriate at the minimum possible value of U_{tot}, which is termed as U_{eq}. The charge $Q^{eq}_{S_1} = -qn^{eq}_{S_1}$, $Q^{eq}_{S_2} = -qn^{eq}_{S_2}$ and $Q^{eq}_{G_1} = -qn^{eq}_{G_1}$ in the dot S_1, S_2 and G_1 respectively, in equilibrium (minimum energy condition) at $0K$, can be calculated by solving the set of equations given below:

$$\frac{\partial U_{tot}}{\partial Q_{S_1}} = \frac{Q_{S_1}}{C^{\Sigma}_{S_1}} + \frac{1}{C^{\Sigma}_{S_1}} (C_{g1,S_1} V_{g1} + C_{S_1,G_1} V_{G_1}) + \frac{(C_{S_1,G_1})^2 Q_{S_1}}{C^{\Sigma}_{G_1} (C^{\Sigma}_{S_1})^2} + \frac{C_{S_1,G_1} Q_{G_1}}{C^{\Sigma}_{S_1} C^{\Sigma}_{G_1}} = 0$$
$$\frac{\partial U_{tot}}{\partial Q_{S_2}} = \frac{Q_{S_2}}{C^{\Sigma}_{S_2}} + \frac{C_{g2,S_2}}{C^{\Sigma}_{S_2}} V_{g2} = 0$$
$$\frac{\partial U_{tot}}{\partial Q_{G_1}} = \frac{Q_{G_1}}{C^{\Sigma}_{G_1}} + \frac{1}{C^{\Sigma}_{G_1}} (C_{g3,G_1} V_{g3} + C_{S_1,G_1} V_{G_3}) + \frac{(C_{S_1,G_1})^2 Q_{G_1}}{C^{\Sigma}_{S_1} (C^{\Sigma}_{G_1})^2} + \frac{C_{S_1,G_1} Q_{S_1}}{C^{\Sigma}_{G_1} C^{\Sigma}_{S_1}} = 0 \tag{4}$$

The above set of equations can be derived by partial differentiation of Eq. 3, and replacing appropriate expressions obtained from partial differentiation and algebraic manipulation of the set of Eqns. 1. The number of electrons in the dots may vary stochastically due to application of external voltage bias or thermal fluctuations from the reservoir at finite temperature. The small increase in the net system electrostatic potential energy due to application of external bias or thermal fluctuations from the reservoirs can be given via a Taylor's expansion of Eq. (3) around the equilibrium dot charges ($-qn^{eq}_{S_1}$, $-qn^{eq}_{S_2}$ and $-qn^{eq}_{G_1}$), along with the condition $\frac{\partial U_{tot}}{\partial Q_{S_1}}\Big|_{Q_{S_1}=-qn^{eq}_{S_1}} = \frac{\partial U_{tot}}{\partial Q_{S_2}}\Big|_{Q_{S_2}=-qn^{eq}_{S_2}} = \frac{\partial U_{tot}}{\partial Q_{G_1}}\Big|_{Q_{G_1}=-qn^{eq}_{G_1}} = 0$ as:

$$U(n_{S_1}, n_{G_1}, n_{S_2}) = U_{tot} - U_{eq} = \sum_{x \in (S_1,G_1,S_2)} \frac{q^2}{C^{self}_x} \left(n^{tot}_x - n^{eq}_x\right)^2 + \sum_{(x_1,x_2) \in (S_1,G_1,S_2)}^{x_1 \neq x_2} U_{x_1,x_2} \left(n^{tot}_{x1} - n^{eq}_{x1}\right)\left(n^{tot}_{x2} - n^{eq}_{x2}\right) \tag{5}$$

where n^{tot}_x is the total number of electrons, and C^{self}_x is self capacitance of the dot x. U_{x_1,x_2} denotes the electrostatic energy arising out of mutual Coulomb coupling between two different quantum dots, which accounts for a fluctuation in the electronic

number of dot $x_{1(2)}$ affecting the electrostatic energy of dot $x_{2(1)}$. These quantities can be derived from the sets of Eqns. (1), (3) and (4), along with the assumption $C_{L,S_1} = C_{R,S_2} = C_{G,G_1} = C_{S_1,S_2} = C_{G_1,S_2} = 0$ as:

$$\frac{1}{C^{self}_{S_1}} = \frac{\partial^2 U_{tot}}{\partial Q^2_{S_1}} = \frac{1}{C^{\Sigma}_{S_1}} + 2\frac{(C_{S_1,G_1})^2}{(C^{\Sigma}_{S_1})^2 C^{\Sigma}_{G_1}}$$
$$\frac{1}{C^{self}_{S_2}} = \frac{\partial^2 U_{tot}}{\partial Q^2_{S_2}} = \frac{1}{C^{\Sigma}_{S_2}}$$
$$\frac{1}{C^{self}_{G_1}} = \frac{\partial^2 U_{tot}}{\partial Q^2_{G_1}} = \frac{1}{C^{\Sigma}_{G_1}} + 2\frac{(C_{S_1,G_1})^2}{(C^{\Sigma}_{G_1})^2 C^{\Sigma}_{S_1}}$$
$$\frac{U_{S_1,G_1}}{q^2} = \frac{\partial^2 U_{tot}}{\partial Q_{G_1}\partial Q_{S_1}} = \frac{\partial^2 U_{tot}}{\partial Q_{S_1}\partial Q_{G_1}} = 2\frac{C_{S_1,G_1}}{C^{\Sigma}_{S_1} C^{\Sigma}_{G_1}}$$

$$\frac{U_{S_1,S_2}}{q^2} = \frac{\partial^2 U_{tot}}{\partial Q_{S_1}\partial Q_{S_2}} = \frac{\partial^2 U_{tot}}{\partial Q_{S_2}\partial Q_{S_1}} = 0$$
$$\frac{U_{S_2,G_1}}{q^2} = \frac{\partial^2 U_{tot}}{\partial Q_{G_1}\partial Q_{S_2}} = \frac{\partial^2 U_{tot}}{\partial Q_{S_2}\partial Q_{G_1}} = 0 \tag{6}$$

From Eq. (5), I proceed to derive the QME of the entire system. In the triple dot set-up, the additional quantum dot S_2 is tunnel coupled to the S_1, while G_1 is Coulomb coupled to S_1. We assume that the electrostatic energy due to self-capacitance is much greater than the average thermal energy or the applied bias voltage, that is $E^{self}_x = \frac{q^2}{C^{self}_x} >> (kT,\ qV)$, such that electronic transport through the Coulomb blocked energy level, due to self-capacitance, can be neglected. Thus the maximum number of electrons in the ground states of each quantum dots is limited to 1. Under all these assumptions, the system analysis may be restricted to $2^3 = 8$ multi-electron states, that I indicate by the electron number in each quantum dot. Thus a state of interest in the system may be denoted by $|n_{S_1}, n_{G_1}, n_{S_2}\rangle = |n_{S_1}\rangle \otimes |n_{G_1}\rangle \otimes |n_{S_2}\rangle$, where $(n_{S_1}, n_{G_1}, n_{S_2}) \in (0,1)$. To simplify these representations of multi-electron states, with a slight abuse of notation, we rename the states as $|0,0,0\rangle \to |0\rangle$, $|0,0,1\rangle \to |1\rangle$, $|0,1,0\rangle \to |2\rangle$, $|0,1,1\rangle \to |3\rangle$, $|1,0,0\rangle \to |4\rangle$, $|1,0,1\rangle \to |5\rangle$, $|1,1,0\rangle \to |6\rangle$, and $|1,1,1\rangle \to |7\rangle$
The simplified Hamiltonian of the triple dot system can hence be written as:

$$H = \sum_{\beta} \varepsilon_\beta |\beta\rangle\langle\beta| + (t\{|3\rangle\langle 6| + |1\rangle\langle 4|\} + h.c.) + U_m\{|6\rangle\langle 6| + |7\rangle\langle 7|\}, \tag{7}$$

where $U_m = U^m_{S_1,G_1}$ is the electrostatic coupling energy between S_1 and G_1 in Supplementary Figure S1, t denotes the interdot tunnel coupling element or hopping parameter between S_1 and S_2 and ε_β is the total energy of the state $|\beta\rangle$ with respect to the vacuum state $|0\rangle$. A mathematical derivation of the form of the Hamiltonian, given in Eqn. (7) may be cumbersome and outside the scope of this paper. We would, however, like to intuitively clarify the Hamiltonian in a step-by-step manner. Let us consider that the three quantum dots S_1, S_2 and G_1 are isolated and there is no hopping element connecting the quantum dots, that is, electrons can't hop from one dot to the other. We aslo initially assume that there is no Coulomb interaction between S_1 and G_1. In this case H is just the sum of the energy of each state multiplied by the projectors of the states. So, in case the three dots are isolated and if there is no Coulomb coupling of tunnel coupling between them, then H is given by $H = H_0 = \sum_\beta \varepsilon_\beta |\beta\rangle\langle\beta|$ In the above expression, ε_β is the total energy of the state $|\beta\rangle$ in the case of zero tunnel and Coulomb coupling. Next, let us consider that we turn on tunnel coupling between the quantum dots. Electrons can tunnel between the dots S_1 and S_2. However, no electrons can tunnel between $S_{1(2)}$ and G_1. Thus, tunneling can take the state $|1,1,0\rangle$ ($|6\rangle$) to $|0,1,1\rangle$ ($|3\rangle$) or vice-versa and $|1,0,0\rangle$ ($|4\rangle$) to $|0,0,1\rangle$ ($|1\rangle$) or vice-versa. Considering the hopping parameter between S_1 and S_2 to be t, the Hamiltonian H now becomes $H = H_0 + H_t$, where $H_t = t|3\rangle\langle 6| + t|1\rangle\langle 4| + h.c.$ So, $H = H_0 + H_t = \Sigma_\beta \varepsilon_\beta |\beta\rangle\langle\beta| + t(|3\rangle\langle 6| + |1\rangle\langle 4| + h.c.)$ In the above expression, h.c. denotes the Hermitian conjugate of the terms $t|3\rangle\langle 6| + t|1\rangle\langle 4|$. Next, we turn on mutual Coulomb coupling between the dots S_1 and G_1. The charging energy U_m due to mutual Coulomb coupling comes into picture only when both S_1 and G_1 are occupied. Thus, the charging energy only occurs when the system is in the state $|6\rangle$ or $|7\rangle$. We would like to state that the state $|6\rangle$ and $|7\rangle$ refers to $|1,1,0\rangle$ and $|1,1,1\rangle$ respectively. In these two cases electrons are present in the ground states of both S_1 and G_1. Due to mutual Coulomb coupling between S_1 and G_1, the energy of these states $|6\rangle$ and $|7\rangle$ should be $\varepsilon_6 + U_m$ and $\varepsilon_7 + U_m$ respectively. So, the term $H_c = U_m(|6\rangle\langle 6| + |7\rangle\langle 7|)$ come into the Hamiltonian to denote the fact the charging energy due to mutual Coulomb coupling gets activated only when the system is in state $|6\rangle$ or $|7\rangle$. Thus, the total Hamiltonian, taking into account tunnel and Coulomb coupling becomes $H = H_0 + H_t + H_c$, which is same as given by Eqn. (7).

Under the assumption that the interdot coupling element t or the reservoir to dot coupling are small, the temporal dynamics of the system density matrix can be evaluated by taking the partial trace over the entire density matrix of the combined set-up consisting of the reservoirs and the dots[10–15]. In this framework, the diagonal and the non-diagonal terms of the triple dot density matrix ρ can be written as a set of modified Liouville equation[10–15]:

$$\begin{aligned}\frac{\partial \rho_{\eta\eta}}{\partial t} &= -i[H,\rho]_{\eta\eta} - \sum_{\nu}\Gamma_{\eta\nu}\rho_{\eta\eta} + \sum_{\delta}\Gamma_{\delta\eta}\rho_{\delta\delta} \\ \frac{\partial \rho_{\eta\beta}}{\partial t} &= -i[H,\rho]_{\eta\beta} - \frac{1}{2}\sum_{\nu}\Big(\Gamma_{\eta\nu}+\Gamma_{\beta\nu}\Big)\rho_{\eta\beta},\end{aligned} \tag{8}$$

where $\rho_{\eta\beta} = \langle\eta|\rho|\beta\rangle$ and $[x,y]$ denotes the commutator of the operators x and y. The terms $\rho_{\eta\eta}$ and $\rho_{\eta\beta}$ in the above equation represent diagonal and non-diagonal elements of the system density matrix respectively. The off-diagonal elements $\rho_{\eta\beta}$ account for coherent inter-dot tunneling, in addition to tunneling of electrons between the dots and the reservoirs. The off-diagonal terms $\rho_{\eta\beta}$, thus, are only non-zero and finite when electron tunneling can result in the transition between the states η and β or vice-versa. The parameters Γ_{xy} account for the transition between system states due to electronic tunneling between the system and the reservoirs and are only finite when the system state transition from $|x\rangle$ to $|y\rangle$ (or vice-versa) is possible due to electron transfer between the system and the reservoirs. Assuming a statistical quasi-Fermi distribution inside the reservoirs, Γ_{xy} can be given as:

$$\Gamma_{xy} = \gamma_\lambda f_\lambda(\varepsilon_y - \varepsilon_x), \tag{9}$$

where $f_\lambda(\varepsilon)$ denotes the probability of occupancy of an electron in the corresponding reservoir λ (driving the state transition) at energy ε, $\varepsilon_{x(y)}$ is the total electronic energy in the state $|x(y)\rangle$ compared to vacuum, and γ_λ denotes the system to reservoir coupling for the corresponding reservoir λ.

For the triple dot set-up, tunneling of electrons between the quantum dots drives the system from $|4\rangle$ to $|1\rangle$ and from $|3\rangle$ to $|6\rangle$ (or vice-versa). In steady state, the time-derivative of each density matrix element $[\rho]$ vanishes. Hence, employing the second equation of (8), we get,

$$\rho_{4,1} = \rho^*_{1,4} = \frac{\rho_{4,4}-\rho_{1,1}}{\varepsilon_4-\varepsilon_1-i\frac{\Upsilon_{4,1}}{2}} \tag{10}$$

$$\rho_{6,3} = \rho^*_{3,6} = \frac{\rho_{6,6}-\rho_{3,3}}{\varepsilon_6-\varepsilon_3-i\frac{\Upsilon_{6,3}}{2}}, \tag{11}$$

where $\Upsilon_{x,y}$ is the sum of net tunneling rates between the system and the reservoirs that leads to the decay of the states $|x\rangle$ and $|y\rangle$. In Eqns. (10) and (11), $\Upsilon_{4,1}$ and $\Upsilon_{6,3}$ are given by:

$$\begin{aligned}\Upsilon_{4,1} &= \Gamma_{|4\rangle,|0\rangle}+\Gamma_{|4\rangle,|6\rangle}+\Gamma_{|4\rangle,|5\rangle}+\Gamma_{|1\rangle,|0\rangle}+\Gamma_{|1\rangle,|6\rangle}+\Gamma_{|1\rangle,|3\rangle} \\ \Upsilon_{6,3} &= \Gamma_{|6\rangle,|4\rangle}+\Gamma_{|6\rangle,|2\rangle}+\Gamma_{|6\rangle,|7\rangle}+\Gamma_{|3\rangle,|1\rangle}+\Gamma_{|3\rangle,|2\rangle}+\Gamma_{|3\rangle,|7\rangle}\end{aligned} \tag{12}$$

From Eq. (8), the time derivative of the density matrix elements $\rho_{6,6}$ and $\rho_{3,3}$ can be given by:

$$\begin{aligned}\dot{\rho}_{6,6} =& it(\rho_{6,3}-\rho_{3,6}) - \Big(\Gamma_{|6\rangle,|4\rangle}-\Gamma_{|6\rangle,|2\rangle}+\Gamma_{|6\rangle,|7\rangle}\Big)\rho_{6,6}+\Gamma_{|4\rangle,|6\rangle}\rho_{4,4}+\Gamma_{|2\rangle,|6\rangle}\rho_{2,2}+\Gamma_{|7\rangle,|6\rangle}\rho_{7,7} \\ \dot{\rho}_{4,4} =& it(\rho_{4,1}-\rho_{1,4}) - \Big(\Gamma_{|4\rangle,|0\rangle}-\Gamma_{|4\rangle,|6\rangle}+\Gamma_{|4\rangle,|5\rangle}\Big)\rho_{4,4}+\Gamma_{|0\rangle,|4\rangle}\rho_{0,0}+\Gamma_{|6\rangle,|4\rangle}\rho_{6,6}+\Gamma_{|5\rangle,|4\rangle}\rho_{5,5}\end{aligned} \tag{13}$$

Substituting the values of $\rho_{6,3}$, $\rho_{3,6}$, $\rho_{4,1}$ and $\rho_{1,4}$ from Eq. (10) and (11) in Eq. (13), time derivative of the probability of the states $|4\rangle$ and $|6\rangle$ can be given by:

$$\dot{p_6} = \dot{\rho}_{6,6} = \sum_{\alpha=}\left(-\Gamma_{|6\rangle,|\alpha\rangle}p_6+\Gamma_{|\alpha\rangle,|6\rangle}p_\alpha\right)-\Lambda_{|6\rangle,|3\rangle}p_6+\Lambda_{|3\rangle,|6\rangle}p_3 \tag{14}$$

$$\dot{p_4} = \dot{\rho}_{4,4} = \sum_{\alpha=}\left(-\Gamma_{|4\rangle,|\alpha\rangle}p_3+\Gamma_{|\alpha\rangle,|4\rangle}p_\alpha\right)-\Lambda_{|4\rangle,|1\rangle}p_4+\Lambda_{|1\rangle,|4\rangle}p_1, \tag{15}$$

where $p_\eta = \rho_{\eta,\eta}$ and

$$\Lambda_{|6\rangle,|3\rangle} = \Lambda_{|3\rangle,|6\rangle} = t^2 \frac{\Upsilon_{6,3}}{(\varepsilon_6 - \varepsilon_3)^2 + \frac{\Upsilon_{6,3}^2}{4}}$$
$$\Lambda_{|4\rangle,|1\rangle} = \Lambda_{|1\rangle,|4\rangle} = t^2 \frac{\Upsilon_{4,1}}{(\varepsilon_4 - \varepsilon_1)^2 + \frac{\Upsilon_{4,1}^2}{4}} \tag{16}$$

In Eq. 16, $\Lambda_{|4\rangle,|1\rangle}$ and $\Lambda_{|6\rangle,|3\rangle}$ denote the rates of interdot tunneling between S_1 and S_2 with empty and occupied ground states of G_1 respectively. By a smart choice of the ground state energy positions, the condition $\varepsilon_6 = \xi_g + \xi_s^1 + U_m = \xi_g + \xi_s^2 = \varepsilon_3$, that is $\xi_s^2 = \xi_s^1 + U_m$ is satisfied. In such a case, under the condition $U_m >> |\Upsilon_{4,1}|$, we get $\Lambda_{|6\rangle,|3\rangle} >> \Lambda_{|4\rangle,|1\rangle}$. This condition implies that the inter-dot tunneling probability between S_1 and S_2 is negligible when the ground state in G_1 is empty.

For the calculation of current, we need to know the probability of ground state in S_1 or S_2. Since, the electronic transport via the ground states in S_1 and G_1 are coupled to each other by Coulomb interaction, we consider S_1 and G_1 as a sub-system (ς_1) of the total triple dot system. S_2 is considered to be the complementary sub-system (ς_2) of the entire set-up. For the range of parameters used in this case, the condition $U_m >> |\Upsilon_{4,1}|$ is satisfied, which leads to $\Lambda_{|4\rangle,|1\rangle} << \Lambda_{|6\rangle,|3\rangle}$. Hence, to simplify the calculations, we assume that $\Lambda_{|4\rangle,|1\rangle} \approx 0$ all practical purposes relating to electron transport. In the following discussion, we simply denote $\Lambda_{|6\rangle,|3\rangle}$ as γ to represent the interdot tunnel coupling. We denote the occupancy probability of the subsystem ς_1 as $P_{i,j}^{\varsigma_1}$, where i and j denote the electron number in the ground state of S_1 and G_1 respectively, while $P_k^{\varsigma_2}$ denotes the ground state occupancy probability of S_2. Note that splitting the entire system into two sub-systems in this fashion demands the limit of weak tunnel and Coulomb coupling between the two sub-systems such that the state of one sub-system remains unaffected by the state of the complementary sub-system. In such a limit, we can write $\rho_{0,0} = P_{0,0}^{\varsigma_1} P_0^{\varsigma_2}$, $\rho_{1,1} = P_{0,0}^{\varsigma_1} P_1^{\varsigma_2}$, $\rho_{2,2} = P_{0,1}^{\varsigma_1} P_0^{\varsigma_2}$, $\rho_{3,3} = P_{0,1}^{\varsigma_1} P_1^{\varsigma_2}$, $\rho_{4,4} = P_{1,0}^{\varsigma_1} P_0^{\varsigma_2}$, $\rho_{5,5} = P_{1,0}^{\varsigma_1} P_1^{\varsigma_2}$, $\rho_{6,6} = P_{1,1}^{\varsigma_1} P_1^{\varsigma_2}$, $\rho_{7,7} = P_{1,1}^{\varsigma_1} P_1^{\varsigma_2}$ The quantum master equations (QME) for the sub-system ς_1 can be given in terms of two or more diagonal elements of the density matrix, in (8), as:

$$\frac{d}{dt}(P_{0,0}^{\varsigma_1}) = \frac{d}{dt}(\rho_{0,0} + \rho_{1,1}) = -P_{0,0}^{\varsigma_1}\{\gamma_l f_L(\xi_s^1) + \gamma_g f_G(\xi_g)\} + \gamma_g P_{0,1}^{\varsigma_1}\{1 - f_G(\xi_g)\} + \gamma_l P_{1,0}^{\varsigma_1}\{1 - f_L(\xi_s^1)\}$$
$$\frac{d}{dt}(P_{1,0}^{\varsigma_1}) = \frac{d}{dt}(\rho_{5,5} + \rho_{4,4}) = -P_{1,0}^{\varsigma_1}\left\{\gamma_l\left(1 - f_L(\xi_s^1)\right) + \gamma_g f_G(\xi_g + U_m)\right\} + \gamma_g P_{1,1}^{\varsigma_1}\left\{1 - f_G(\xi_g + U_m)\right\} + \gamma_g P_{0,0}^{\varsigma_1} f_L(\xi_s^1)$$
$$\frac{d}{dt}(P_{0,1}^{\varsigma_1}) = \frac{d}{dt}(\rho_{2,2} + \rho_{3,3}) = -P_{0,1}^{\varsigma_1}\left\{\gamma_g\left(1 - f_g(\xi_g^1)\right) + \gamma_l f_L(\xi_s^1 + U_m) + \gamma P_1^{\varsigma_2}\right\}$$
$$+ \gamma_g P_{0,0}^{\varsigma_1} f_G(\xi_g) + P_{1,1}^{\varsigma_1}\left\{\gamma_l\left(1 - f_L(\xi_s^1 + U_m)\right) + \gamma P_0^{\varsigma_2}\right\}$$
$$\frac{d}{dt}(P_{1,1}^{\varsigma_1}) = \frac{d}{dt}(\rho_{7,7} + \rho_{6,6}) = -P_{1,1}^{\varsigma_1}\left\{\gamma_g\left(1 - f_g(\xi_g^1 + U_m)\right) + \gamma_l\left(1 - f_L(\xi_s^1 + U_m)\right) + \gamma P_0^{\varsigma_2}\right\}$$
$$+ \gamma_g P_{1,0}^{\varsigma_1} f_G(\xi_g + U_m) + P_{0,1}^{\varsigma_1}\left\{\gamma_l f_L(\xi_s^1 + U_m) + \gamma P_1^{\varsigma_2}\right\} \tag{17}$$

where $\gamma = \Lambda_{|6\rangle,|3\rangle} = \Lambda_{|3\rangle,|6\rangle}$ and $\Lambda_{|4\rangle,|1\rangle} = \Lambda_{|1\rangle,|4\rangle} = 0$. I assume quasi Fermi-Dirac electron distribution at the reservoirs. Hence, corresponding to the reservoir λ, and $\lambda \in (L,R,G)$ $f_\lambda(\varepsilon) = \left\{1 + exp\left(\frac{\varepsilon - \mu_\lambda}{kT_\lambda}\right)\right\}^{-1}$. Similarly, the QME of the sub-system ς_2 can be written as:

$$\frac{d}{dt}(P_0^{\varsigma_2}) = \frac{d}{dt}(\rho_{6,6} + \rho_{4,4} + \rho_{2,2} + \rho_{0,0}) = -P_0^{\varsigma_2}\{\gamma_r f_R(\xi_s^2) + \gamma P_{1,1}^{\varsigma_1}\} + P_1^{\varsigma_2}\{\gamma_r\left(1 - f_R(\xi_s^2)\right) + \gamma P_{0,1}^{\varsigma_1}\}$$
$$\frac{d}{dt}(P_1^{\varsigma_2}) = \frac{d}{dt}(\rho_{7,7} + \rho_{5,5} + \rho_{3,3} + \rho_{1,1}) = -P_1^{\varsigma_2}\{\gamma_r\left(1 - f_R(\xi_s^2)\right) + \gamma P_{0,1}^{\varsigma_1}\} + P_0^{\varsigma_2}\{\gamma_r f_R(\xi_s^2) + \gamma P_{1,1}^{\varsigma_1}\} \tag{18}$$

The L.H.S of Eqns. (17) and (18) are zero in steady state. The set of Eqns. (17) and (18) form a coupled system of equations which were solved iteratively via Newton-Raphson method. On solution of the steady-state probabilities, the charge current $I_{L(R)}$ between reservoir L and R and the electronic heat current (I_{Qe}) extracted from the reservoir G can be calculated by the equations:

$$I_L = q\gamma_l \times \left\{P_{0,0}^{\varsigma_1} f_L(\xi_s^1) + P_{0,1}^{\varsigma_1} f_L(\xi_s^1 + U_m) - P_{1,0}^{\varsigma_1}\{1 - f_L(\xi_s^1)\} - P_{1,1}^{\varsigma_s^1}\{1 - f_L(\xi_s^1 + U_m)\}\right\} \tag{19}$$

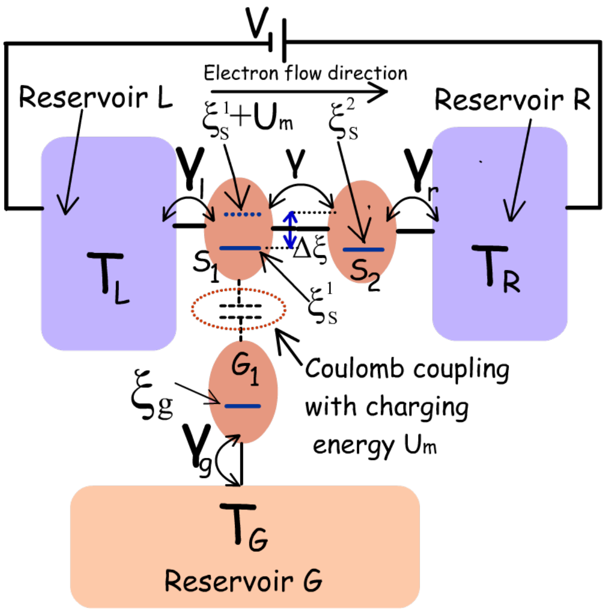

Supplementary Figure S3: Schematic diagram for an equivalent triple dot design to accomplish efficient non-local thermometry. The thermometry performance of this set-up was found to be similar to the proposed triple dot set-up in Supplementary Figure S1(b) with a different regime of operation.

$$I_R = -q\gamma_r \times \left\{P_0^{\varsigma 2} f_R(\xi_s^1) - P_1^{\varsigma 2}\{1 - f_R(\xi_s^1)\}\right\} \tag{20}$$

$$I_Q = \gamma_g \times \left\{(\xi_g + U_m - \mu_g)\left\{P_{1,0}^{\varsigma 1} f_G(\xi_g + U_m) - P_{1,1}^{\varsigma 1}\{1 - f_G(\xi_g + U_m)\}\right\}\right\}$$
$$+\gamma_g \times \left\{(\xi_g - \mu_g) \times \left\{P_{0,0}^{\varsigma 1} f_G(\xi_g) - P_{0,1}^{\varsigma n}\{1 - f_G(\xi_g)\}\right\}\right\} \tag{21}$$

Since, net current into (out-of) the reservoir G is zero, we have

$$I_G = q\gamma_g \times \left\{P_{1,0}^{\varsigma 1} f_G(\xi_g + U_m) - P_{1,1}^{\varsigma 1}\{1 - f_G(\xi_g + U_m)\} + P_{0,0}^{\varsigma 1} f_G(\xi_g) - P_{0,1}^{\varsigma n}\{1 - f_G(\xi_g)\}\right\} = 0 \tag{22}$$

Substituting Eq. (22) in Eq. (21), we get

$$I_Q = \gamma_g \times U_m \left\{P_{1,0}^{\varsigma 1} f_G(\xi_g + U_m) - P_{1,1}^{\varsigma 1}\{1 - f_G(\xi_g + U_m)\}\right\} \tag{23}$$

3 Another equivalent triple dot set-up for efficient non-local thermometry

In this section, we show a variant of the triple dot set-up that can also be employed for efficient non-local thermometry in the regime of hundreds of "milli-Kelvin". This set-up is demonstrated in Supplementary Figure S3 and is identical in construction to the set-up investigated in this paper (in Fig. 1.b) in the main text. However, unlike the proposed set-up in Fig. 1(b) in the main text, the ground-states of the two quantum dots S_1 and S_2 are aligned with each other, that is $\xi_s^1 = \xi_s^2$. In this case, an electron tunneling from G into G_1 misaligns the ground states in S_1 and S_2 and blocks the current flow through the system. A change in temperature of the reservoir G impacts the probability of occupancy of G_1 and thus induces thermometry. Although not elaborated here, the configuration demonstrated in Supplementary Figure S3 demonstrates similar thermometry performance to the set-up shown in Fig. 1(b), in the main text, with a different regime of operation. The configuration, demonstrated in Supplementary Figure S3, thus provides an alternative arrangement for efficient non-local thermometry.

References

1. Zhang, Y. & Chen, J. Thermometry based on coulomb-coupled quantum dots. *Phys. E: Low-dimensional Syst. Nanostructures* **114**, 113635, DOI: https://doi.org/10.1016/j.physe.2019.113635 (2019).

2. Sánchez, R. & Büttiker, M. Optimal energy quanta to current conversion. *Phys. Rev. B* **83**, 085428, DOI: 10.1103/PhysRevB.83.085428 (2011).
3. Thierschmann, H. *et al.* Three-terminal energy harvester with coupled quantum dots. *Nat. Nanotechnol.* **10**, 854–858, DOI: 10.1038/nnano.2015.176 (2015).
4. Andreas, F. & Carina, F. Coulomb blockade in quantum dots, DOI: http://www.ftf.lth.se/fileadmin/ftf/Course_pages/FFF042/cb_lecture07.pdf (2007).
5. Hübel, A., Weis, J., Dietsche, W. & Klitzing, K. v. Two laterally arranged quantum dot systems with strong capacitive interdot coupling. *Appl. Phys. Lett.* **91**, 102101, DOI: 10.1063/1.2778542 (2007).
6. Chan, I. H., Westervelt, R. M., Maranowski, K. D. & Gossard, A. C. Strongly capacitively coupled quantum dots. *Appl. Phys. Lett.* **80**, 1818–1820, DOI: 10.1063/1.1456552 (2002).
7. Molenkamp, L. W., Flensberg, K. & Kemerink, M. Scaling of the coulomb energy due to quantum fluctuations in the charge on a quantum dot. *Phys. Rev. Lett.* **75**, 4282–4285, DOI: 10.1103/PhysRevLett.75.4282 (1995).
8. Hübel, A., Held, K., Weis, J. & v. Klitzing, K. Correlated electron tunneling through two separate quantum dot systems with strong capacitive interdot coupling. *Phys. Rev. Lett.* **101**, 186804, DOI: 10.1103/PhysRevLett.101.186804 (2008).
9. Ruzin, I. M., Chandrasekhar, V., Levin, E. I. & Glazman, L. I. Stochastic coulomb blockade in a double-dot system. *Phys. Rev. B* **45**, 13469–13478, DOI: 10.1103/PhysRevB.45.13469 (1992).
10. Gurvitz, S. A. Rate equations for quantum transport in multidot systems. *Phys. Rev. B* **57**, 6602–6611, DOI: 10.1103/PhysRevB.57.6602 (1998).
11. Hazelzet, B. L., Wegewijs, M. R., Stoof, T. H. & Nazarov, Y. V. Coherent and incoherent pumping of electrons in double quantum dots. *Phys. Rev. B* **63**, 165313, DOI: 10.1103/PhysRevB.63.165313 (2001).
12. Dong, B., Lei, X. L. & Horing, N. J. M. First-order coherent resonant tunneling through an interacting coupled-quantum-dot interferometer: Generic quantum rate equations and current noise. *Phys. Rev. B* **77**, 085309, DOI: 10.1103/PhysRevB.77.085309 (2008).
13. Dong, B., Cui, H. L. & Lei, X. L. Quantum rate equations for electron transport through an interacting system in the sequential tunneling regime. *Phys. Rev. B* **69**, 035324, DOI: 10.1103/PhysRevB.69.035324 (2004).
14. Sztenkiel, D. & Świrkowicz, R. Electron transport through parallel double quantum dots with interdot correlations. *physica status solidi (b)* **244**, 2543–2548, DOI: 10.1002/pssb.200674623 (2007).
15. Wegewijs, M. R. & Nazarov, Y. V. Resonant tunneling through linear arrays of quantum dots. *Phys. Rev. B* **60**, 14318–14327, DOI: 10.1103/PhysRevB.60.14318 (1999).
